# Characterizing and mitigating confounding by unreplicated evolutionary events in phylogenetic regression

**DOI:** 10.64898/2025.12.04.692436

**Published:** 2025-12-05

**Authors:** Qifan Wang, Michael D. Edge, Joshua G. Schraiber, Matt Pennell

**Affiliations:** 1 Department of Computational Biology, Cornell University; 2 Department of Quantitative and Computational Biology, University of Southern California

## Abstract

When researchers want to test for a direct effect of one attribute on another using interspecific data, they need to account for the confounding influence of other attributes that have phylogenetic structure. Accordingly, a suite of approaches have been developed for this purpose, mostly notably the use of phylogenetic regression. However, it has been shown that such methods can be misled by evolutionary events with large effects. We suggest that one widely-applicable solution to this problem can be found by borrowing both from the statistical genetics literature and by digging into the past of phylogenetic comparative methods. In conventional phylogenetic regression, the phylogenetic variance-covariance matrix is included as a random-effect term; here, we argue that it can be advantageous to also include the leading eigenvectors of this matrix as fixed effects. Building on the recent results of Schraiber et al. (2024), we use mathematical analysis and simulations to show under which scenarios this hybrid strategy is effective. We develop a novel approach for visualizing the contributions of different branches of the phylogeny to the eigenvectors of the variance-covariance matrix. We also illustrate how quantile-quantile plots can be used to assess whether the phylogenetic structure has been effectively controlled. We then apply the mixed approach to investigate the co-evolution of gene expression levels of different genes across the phylogeny of cichlid fishes; we show that including the leading eigenvectors likely reduces the false positive rate. To facilitate the use of our visualization approach and of including eigenvectors in phylogenetic regression models, we present a new R package called EIGER. We argue that the approaches we explore here can both help address the statistical problems that arise from large, historical events and more generally, provide richer insights into the nature of phylogenetic confounding.

## Introduction

It is widely appreciated that correcting for phylogenetic structure is a critical requirement in many, and perhaps most, hypothesis tests involving interspecific data. Indeed, it is so routine in comparative studies that the debates about the validity and purpose of this approach that occurred decades ago (see, for instance, discussions in Harvey et al., 1991) may now seem obscure. The standard conceptual model is that unmeasured traits affect both the response and explanatory variable (i.e., these traits are confounders) and that the unmeasured traits are distributed in a way that reflects phylogeny (Rohlf, 2001, 2006; Uyeda et al., 2018; Westoby et al., 2023). Phylogenetically indepedent contrasts (Felsenstein, 1985), phylogenetic generalized least squares (Grafen, 1989; Martins and Hansen, 1997) or phylogenetic linear mixed models (Lynch, 1991; Housworth et al., 2004) are the workhorse statistical approaches to model this scenario (for simplicity, we will hereafter use the shorthand ‘PGLS’ to refer to these approaches collectively; as we discuss later, the differences are not pertinent to the present study).

However, as pointed out by Uyeda et al. (2018), PGLS, like many other phylogenetic comparative methods (Read and Nee, 1995; Maddison and FitzJohn, 2015), have unacceptably high false-positive rates when the evolution of traits is characterized by large, rare events that are inconsistent with the continuous processes that are assumed by nearly all models. This is unfortunate for two reasons. First, it is correcting for these large events—the type of evolutionary event that define what taxonomists consider a clade—that is often the stated rationale for the use of phylogenetic comparative methods in the first place (Uyeda et al., 2018). Second, large bursts of trait change have been found to be a characteristic dynamic of many phenotypes of interest (Uyeda et al., 2011; Pennell et al., 2014; Landis and Schraiber, 2017).

There have been several suggestions as to what to do about the pernicious influence of unreplicated events in phylogenetic comparative studies. Uyeda et al. (2018) pointed out a conceptually simple approach: if, for example, there had been only one large shift in the mean phenotype over evolutionary time and it was known where on the phylogeny it occurred, one could simply include an indicator variable in the regression model specifying on which side of the shift event a species falls on the phylogeny. In practice, however, this requires a separate method for identifying the shift point (we return to this problem below). Further, it is unclear how best to construct the model if there are multiple mean shifts along the phylogenetic tree. Hidden Markov models have become widely used in phylogenetic comparative studies to mitigate the influence of unmodeled background variation in evolutionary rates in discrete characters (Beaulieu et al., 2013; Caetano et al., 2018); it is not apparent how to translate this idea into the formulation of a generalized regression model. Last, some researchers have explored the use of robust regression techniques (Slater and Pennell, 2014; Adams et al., 2024, 2025); robust regression methods effectively down-weight high-leverage observations, reducing the false positives that result from large-effect, unreplicated events. Although applying robust regression may be effective in some contexts, these approaches cannot guarantee consistent estimation of variance components and lack some of the flexibility of generalized linear models. Moreover, it is unknown how to map the statistical model used in robust regression to any particular evolutionary model, and thus it is hard to gain intuition as to how and why it may work in any given scenario.

Schraiber et al. (2024) showed that both the PGLS model and the linear mixed model (LMM) used in genome-wide association studies (GWAS) can be derived as a special cases of the same general model. This result suggests that tools developed in one field can often be readily applied in the other. This is valuable because GWAS studies are susceptible to the same type of large-effect events as phylogenetic models. In the context of GWAS, researchers are trying to control the confounding influence of population structure (Freedman et al., 2004; Marchini et al., 2004; Helgason et al., 2005; Campbell et al., 2005); as in phylogenetic comparative methods, the LMM used by statistical geneticists includes random effects that are assumed to be distributed in a way that mirrors the relatedness among individuals in the sample (Kang et al., 2008, 2010; Astle and Balding, 2009; Zhou and Stephens, 2012). A fairly common practice in statistical genetics is to use models that include both random effects parameterized by a relatedness matrix and fixed effects for individuals’ coordinates on the eigenvectors of the same relatedness matrix. Although the apparent redundancy of this “belt and suspenders” approach has been recognized in the GWAS literature, it is commonly used and helpful when large environmental effects covary with population structure (Schraiber et al., 2024).

This state of affairs led us to reason that the solution devised by statistical geneticists could be applied to the case of phylogenetic regression: in addition to including the phylogenetic structure as a random effect in the regression, also include several eigenvectors of the phylogenetic variance co-variance matrix (defined below) as fixed-effect terms. In the 1990s, the idea of using phylogenetic eigenvectors was presented as an alternative to the use of mixed models (Diniz-Filho et al., 1998, referred to as Phylogenetic Eigenvector Regression, or PVR); this approach was argued to have inferior statistical properties to PGLS (Rohlf, 2001; Freckleton et al., 2011). We will discuss the reasons for this assessment below. The insight of statistical geneticists is that one could profitably combine these two strategies (i.e., use both LMMs *and* eigenvectors). This mixed strategy was suggested by Schraiber et al. (2024) and used by David et al. (2025) in a recent study to uncover the genetic bases of convergent metabolic innovation across yeasts. To our knowledge, this idea has not been used elsewhere in the context of comparative biology.

We refer readers to our previous paper (Schraiber et al., 2024) for a more complete theoretical analyses of the use of eigenvectors in the context of a PGLS model, but we repeat the key elements of the approach here. In a sample of size n tips, we have a predictor, $x={\left(x_{1},x_{2},\ldots ,x_{n}\right)}^{T}$ and an outcome variable, $y={\left(y_{1},y_{2},\ldots ,y_{n}\right)}^{T}$, both of which are either organismal traits or proxies thereof. To analyze the effect of x on y, we can use the following regression model:

(1) $$y_{i}=x_{i}\beta +G_{i}+E_{i},$$

where β is the effect of x on y and $G_{i}$ and $E_{i}$ are genetic and environmental components of variance in y, respectively. By genetic component, we are referring to features encoded by the genome of a species aside from x that might influence y. The environmental component refers to non-genetic contributions to the phenotype. In this formulation, the model ignores G×E interactions, following typical practice. Testing for an effect of x on y requires accounting for covariance in the genetic component G, or else hypothesis tests will not have the desired type-I error rate (Rohlf, 2001, 2006). The $G_{i}$ values of different species covary owing to shared ancestry, and we can express the expected pattern of covariation with the “phylogenetic variance-covariance matrix”, which will hereafter denote as Σ. Making standard distributional assumptions, the PGLS model can be written as

(2) $$y\sim \text{Multivariate Normal}\left(\beta x,\sigma_{G}^{2}\text{Σ}+\sigma_{E}^{2}I\right).$$

We can modify this model by including eigenvectors of Σ as fixed effects alongside β. To do so, we perform the eigenvector decomposition $\text{Σ}=V\text{Λ}V^{T}$, where $V=\left[\begin{array}{llll} v_{1} & v_{2} & \cdots & v_{n} \end{array}\right]$ is a matrix containing the eigenvectors of Σ as its columns, and $\text{Λ}=\mathrm{diag}\left(\lambda_{1},\lambda_{2},\ldots ,\lambda_{n}\right)$ is a diagonal matrix with the eigenvalues of Σ on the diagonal. As a variance-covariance matrix, Σ is positive semidefinite, and its eigenvectors can be used to form an orthonormal basis of ${\mathbb{R}}^{n}$.

Schraiber et al. (2024) provided a mathematical argument that using the eigenvectors of Σ as fixed effects in a generalized least squares framework could provide the benefits of both approaches: if an eigenvector of Σ contains some large confounding that is out of proportion with its associated eigenvalue, that confounding can be removed by including the eigenvector in the design matrix. For eigenvectors that are not included in the design matrix, we can still control for spurious association between x and y caused by the phylogenetic structure by utilizing PGLS. Although Schraiber et al. (2024) showed why this approach could work, it was a small part of an argument targeted elsewhere. Furthermore, they focused primarily on the theoretical issues around how eigenvectors capture different components of the variance from PGLS and not on the performance and use of the approach. Here, we perform a much more extensive set of simulations that evaluate the performance of the method in a wider variety of evolutionary scenarios; develop new mathematical results, and an accompanying visualization technique, that illustrate how the eigenvectors capture phylogenetic structure in specific cases; and illustrate the use of quantile-quantile plots for assessing how effectively researchers have removed the influence of phylogenetic confounding. We also discuss pragmatic issues in the application of this technique, address previously raised critiques of phylogenetic eigenvector regression, and provide a new R package EIGER, that implements the approaches described here.

## Methods

### Visualization of contributions of branches to eigenvectors

Before describing the method of visualizing contributions of branches to eigenvectors, we make one note of clarification: our approach is distinct from the computation of “phylogenetic principal components analysis (pPCA)” (Revell, 2009; Polly et al., 2013). In that case, one is computing the eigenvectors of a matrix of traits X and using the Σ matrix to remove the phylogenetic signal. This is mathematically and conceptually distinct from what we are doing here and is used to solve a different problem (see Uyeda etal., 2015; Bookstein, 2019).

**Fig 1:**
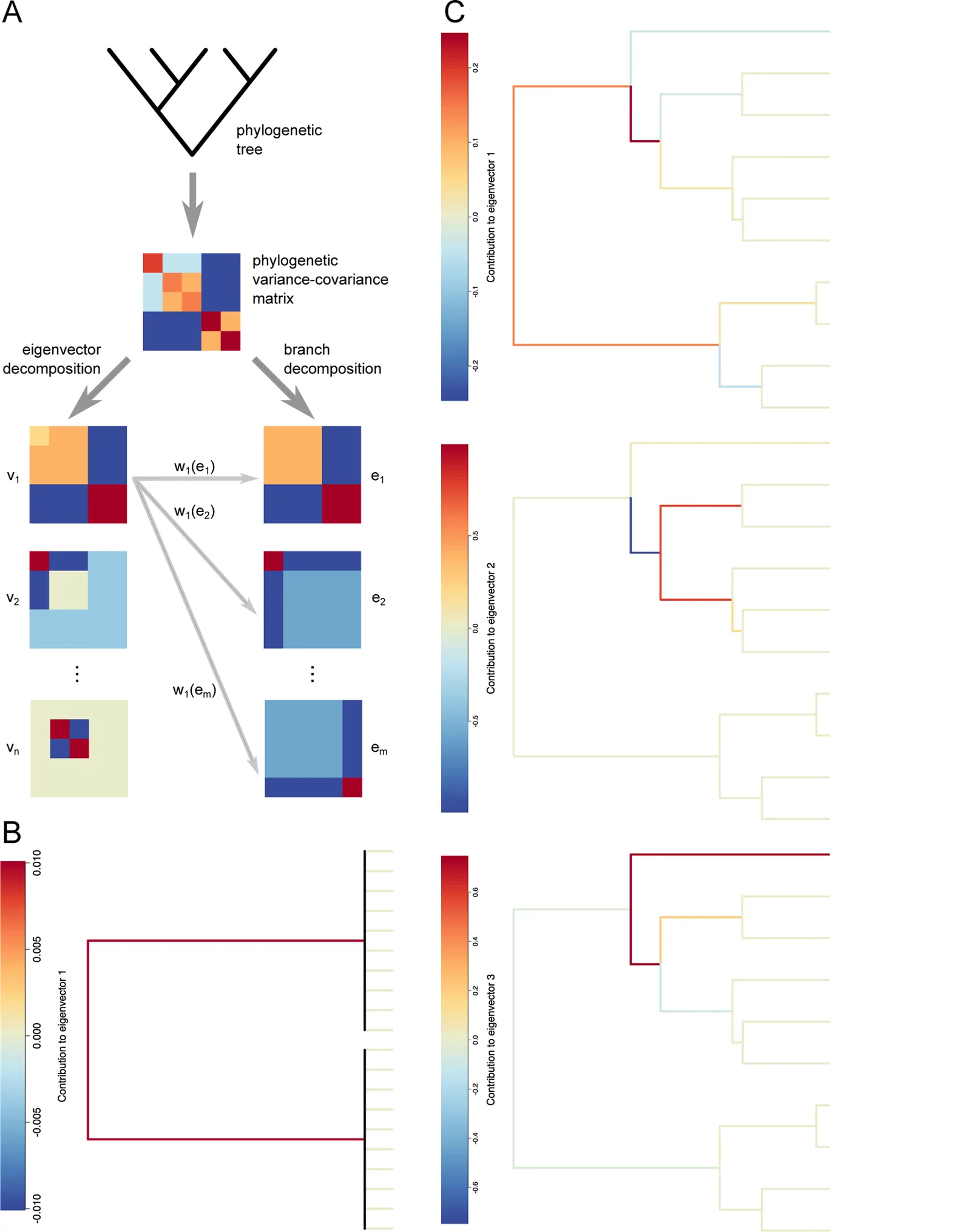
Contributions of branches to the eigenvectors of a phylogenetic variance-covariance matrix Σ. (A) Flowchart showing the method to calculate the contributions of branches to the eigenvectors of Σ. (B) Contributions of branches to $v_{1}$ of the double-centered phylogenetic variance-covariance matrix $\overset{⌣}{C}$ of a phylogenetic tree depicting Felsenstein’s Worst Case (FWC). Each clade contains 10 species. The vertical branches are black because the tip branches slightly differ in the their contributions to $v_{1}$. (C) Contributions of branches to the first three eigenvectors of $\overset{⌣}{C}$ of a Yule tree containing 10 species.

To visualize how eigenvectors capture the phylogenetic structure, we developed a tool to compute the contributions of specific branches of the species tree to the eigenvectors of the phylogenetic variance-covariance matrix (Figure 1A). Population and statistical geneticists have developed the idea of the expected genetic relatedness matrix given a gene-genealogical tree relating haplotypes and assumptions about the mutation process (McVean, 2009; Fan et al., 2022; Zhang et al., 2023; Lehmann et al., 2025), variously called an expected genetic-relatedness-matrix (eGRM), an ancestral-recombination-graph GRM, or a branch GRM; we use the term eGRM to refer to all of these approaches. These eGRMs can be expressed as a weighted sum of eGRMs corresponding to each branch of the tree (Fan et al., 2022). Although the eGRM approach was designed in the context of genealogical trees, the principle applies to phylogenetic trees just as well. Here we extend this idea to our Σ matrix, assuming that traits evolve following a Brownian motion (BM) model across the phylogenetic tree. We denote this particular version of Σ as C. Given the phylogenetic tree T and the BM diffusion rate of $\sigma^{2}$, and the contribution of a branch e to C is

(3) $$C(e)=\sigma^{2}l(e)x(e)x{\left(e\right)}^{T},$$

where l(e) is the branch length, and x(e) is a binary vector for the descendancy relationship, with x(e)[i] being 1 if the *i*-the tip is a descendant of branch e and 0 otherwise. Then, $x(e)x{\left(e\right)}^{T}$ is a matrix where the i, j entry is 1 if both species i and j descend from e. We can compute C for the entire tree T by summing the contributions from all branches:

(4) $$C(T)=\sum_{e\in T}C(e).$$

Note that this equation will result in exactly the variance-covariance matrix in the standard Brownian motion model: the i, j entry will be the sum of branch length share by species i and j scaled by the rate of Brownian motion, $\sigma^{2}$.

The next step would be to perform an eigenvector decomposition. Although we can decompose C itself, in principle, it would be best practice to first perform a double-centering transformation. This transformation can be interpreted as unrooting the tree so that the contributions coming from the two branches stemming from the root would merge into one. This is helpful for visualization, since the partition of the species into descendants and non-descendants of the two branches would be the same: if a species is a descendant of one branch, than it is a non-descendant for the other. This double-centering transformation also helps build the connections between our methods and many previous methods using PCs for controlling confounding in both statistical genetics and phylogenetics contexts (Diniz-Filho et al., 1998; McVean, 2009; de Vienne et al., 2011). We can show that the eigenvectors of the double-centered version of the matrix, which we denote as $\overset{⌣}{C}$, are exactly the same as those of the double-centered distance matrix $\overset{⌣}{D}$ used in Diniz-Filho et al. (1998), with eigenvalues differing by a constant (see Section 3 of the supplementary material). Following de Vienne et al. (2011), we can also show that the eigenvectors of $\overset{⌣}{C}$ correspond to the projections of the species onto the primary axes of a PCA, if we consider the n tips of the phylogenetic tree as n points in ${\mathbb{R}}^{n}$ space (see Section 4 of the supplementary material). Due to the convenient properties of double-centeredness, the usage of a double-centered matrix is even more prevalent in statistical genetics, where the data are essentially always mean-centered column-wise before being used to construct a GRM (see Yang et al., 2010) or eGRM (see Fan et al., 2022), which are statistical genetics equivalents of C. However, we’d also like to note that even though it is theoretically preferable to use the double-centered version $\overset{⌣}{C}$ to get eigenvectors, in practice using a non-centered C would not change the results by much when doing phylogenetic regressions (see Section 5 of the supplementary material for theoretical analyses and Fig. S4, S5, and S14 for simulation results). If we perform a double-centering transformation on C, then we obtain:

(5) $$\overset{⌣}{C}(T)=\left(I-\frac{1}{n}11^{T}\right)C(T)\left(I-\frac{1}{n}11^{T}\right).$$

The eigenvector decomposition of $\overset{⌣}{C}$ is:

(6) $$\overset{⌣}{C}(T)=V\text{Λ}V^{T}=\sum_{d}\lambda_{d}v_{d}v_{d}^{T},$$

where the summand $\lambda_{d}v_{d}v_{d}^{T}$ in the Eq. 6 refers to the contribution from one of the orthogonal dimensions to $\overset{⌣}{C}(T)$. Finally, we can obtain the contributions of branches to the eigenvectors by running a linear regression:

(7) $$v_{d}v_{d}^{T}=\sum_{e\in T}w_{d}(e)\overset{⌣}{C}(e),$$

where $w_{d}(e)$ is the contribution of branch e to the *d*-th eigenvector of the phylogenetic tree. Note that $w_{d}(e)$ does not necessarily have to be positive, and we only care about the relative weights across the branches. Thus $\lambda_{d}$ does not affect the relative weights of Σ(e) here: including $\lambda_{d}$ into the equation would multiply the weights w(e) by a constant factor of $\lambda_{d}$. Once the contributions of branches to each of the eigenvectors are obtained, one can also compute the overall contributions of branches, which can then be used as a metric for the importance of the branches (see Section 6 of the supplementary material).

### Software implementation

We have developed a new open-source R package EIGER that includes two key capabilities. First, using the functions compute_branch_contributions() and plot_branch_contributions(), researchers can evaluate the contributions of different branches on a phylogenetic tree to its eigenvectors. Second, we have built a wrapper for the core function of phylolm (Ho et al., 2016) so that researchers can include eigenvectors as fixed effects in the general PGLS model. This package is available on GitHub https://github.com/applied-phylo-lab/eiger.

### Simulation analysis

**Fig 2:**
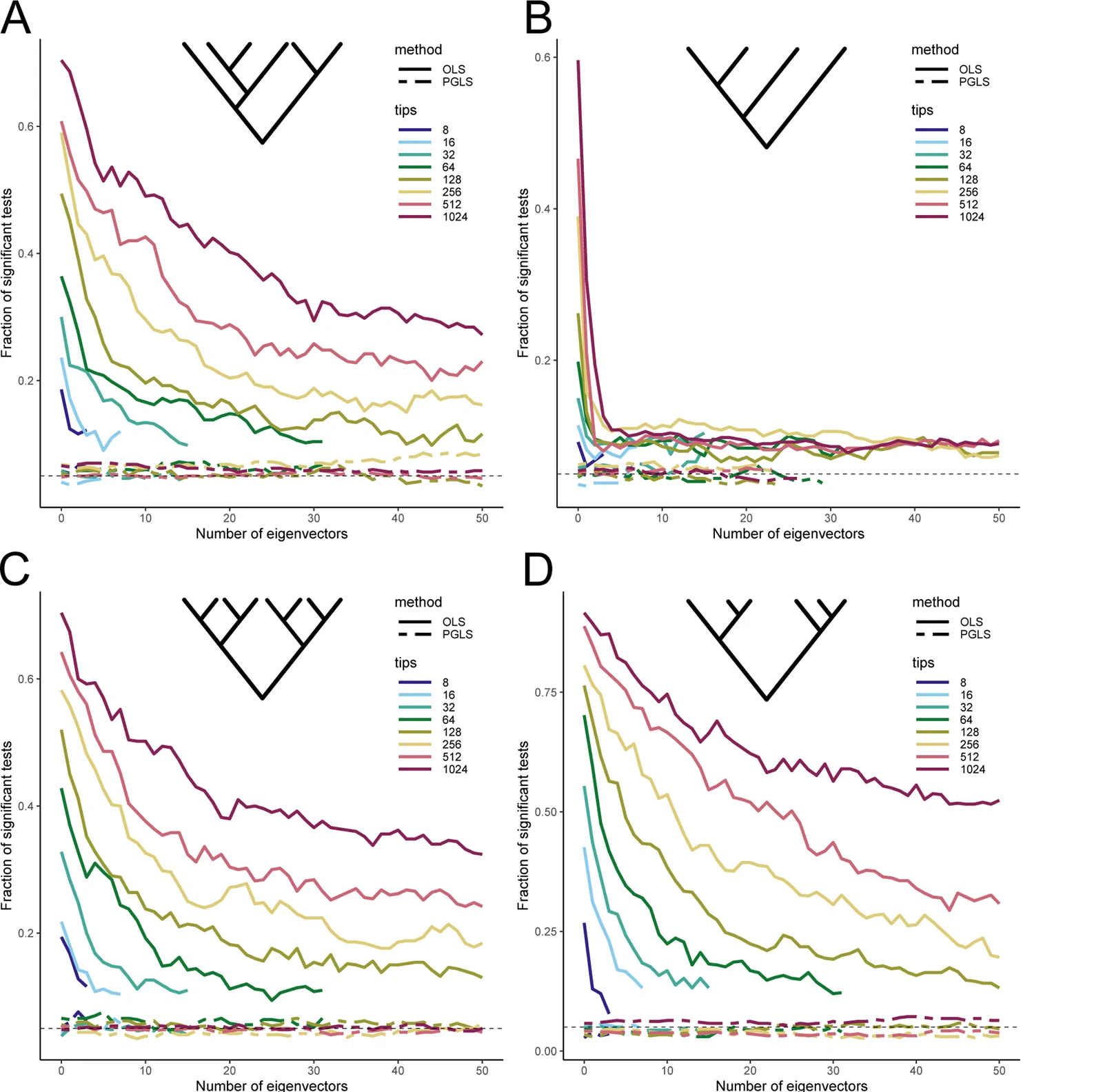
The false positive rate after adding eigenvectors in OLS and PGLS for different types of trees when the data is generated by a pure Brownian motion with no additional shifts. (A) Performance of OLS and PGLS in models related by a Yule tree of varying numbers of species. For simplicity, a Yule tree with only 6 tips is shown. The horizontal axis shows the number of eigenvectors included as covariates, and the vertical axis shows the fraction of tests that would be significant at the 0.05 level. (B) Performance of OLS and PGLS in models related by a caterpillar tree of varying numbers of species. For simplicity, a caterpillar tree with only 4 tips is shown. The horizontal axis shows the number of eigenvectors included as covariates, and the vertical axis shows the fraction of tests that would be significant at the 0.05 level. (Note that when a very large number of eigenvectors were included, the PGLS model could not be estimated.) (C) Performance of OLS and PGLS in models related by a fully balanced tree of varying numbers of species. For simplicity, a fully balanced tree with only 8 tips is shown. The horizontal axis shows the number of eigenvectors included as covariates, and the vertical axis shows the fraction of tests that would be significant at the 0.05 level. (D) Performance of OLS and PGLS in models related by a coalescent tree of varying numbers of species. For simplicity, a coalescent tree with only 6 tips is shown. The horizontal axis shows the number of eigenvectors included as covariates, and the vertical axis shows the fraction of tests that would be significant at the 0.05 level.

Schraiber et al. (2024) investigated the performance of using eigenvectors in PGLS in simple scenarios. In particular, they studied Felsenstein’s Worst Case (hereafter FWC), a scenario developed by Felsenstein (1985) and examined in more detail in Uyeda et al. (2018), in which the only phylogenetic structure in a dataset is between two clades, and there is a shift in the mean trait values along a branch leading to one of the clades; additionally they considered the case of BM evolved on a Yule tree. Here, we are exploring a much larger tree space by considering different tree topologies of varying sizes. To test the effect of tree size, we simulated Yule trees, caterpillar trees, fully balanced trees, and coalescent trees of 8, 16, 32, 64, 128, 256, 512, and 1024 species (Fig. 2). For each combination of tree type and tree size, we generated 500 different ultrametric trees with varying branch lengths. Then we simulated x and y as arising independently from a Brownian motion along each of the trees. For simulations, we used $\sigma_{G}^{2}=1$ and $\sigma_{E}^{2}=0$. Although real datasets feature nontrivial contributions from environmental noise (for further details, see Hansen and Bartoszek, 2012; Silvestro et al., 2015; Pennell et al., 2015), the results would be qualitatively similar (Schraiber et al., 2024). After x and y were generated, we compared the performance of using different numbers of eigenvectors in OLS and PGLS for phylogenetic regression using the functions lm() and phylolm() in the phylolm R package (Ho and Ané, 2014; Ho et al., 2016). In Section 7 of the supplementary material, we show that our results apply equally to PGLS and PGLMM formulations of phylogenetic regression.

We then explored the performance of the methods when there are non-Brownian shifts in trait value on the trees, generalizing the work of Schraiber et al. (2024), who examined FWC with one shift on one of the clades. Here, we revisited the same scenario with a quantile-quantile plot (QQ plot) of *p*-values. If the null hypothesis is true (i.e., there is no association between traits), the *p*-values will follow the expected distribution and they will fall on the 1:1 line of the QQ plot; by convention, we use $-\log_{10}(p)$, which will be uniformly distributed ∈[0,1]. If there are some $-\log_{10}(p)$ that are bigger than expected, this may indicate a true signal of an association; if the entire distribution of $-\log_{10}(p)$ is inflated, this likely means that the phylogenetic structure is confounding the tests. Although this is a routine procedure in statistical genetics (in a GWAS, there will always be many tests performed), we are only aware of one application of this idea to phylogenetically structured data (Hale et al., 2025).

**Fig 3:**
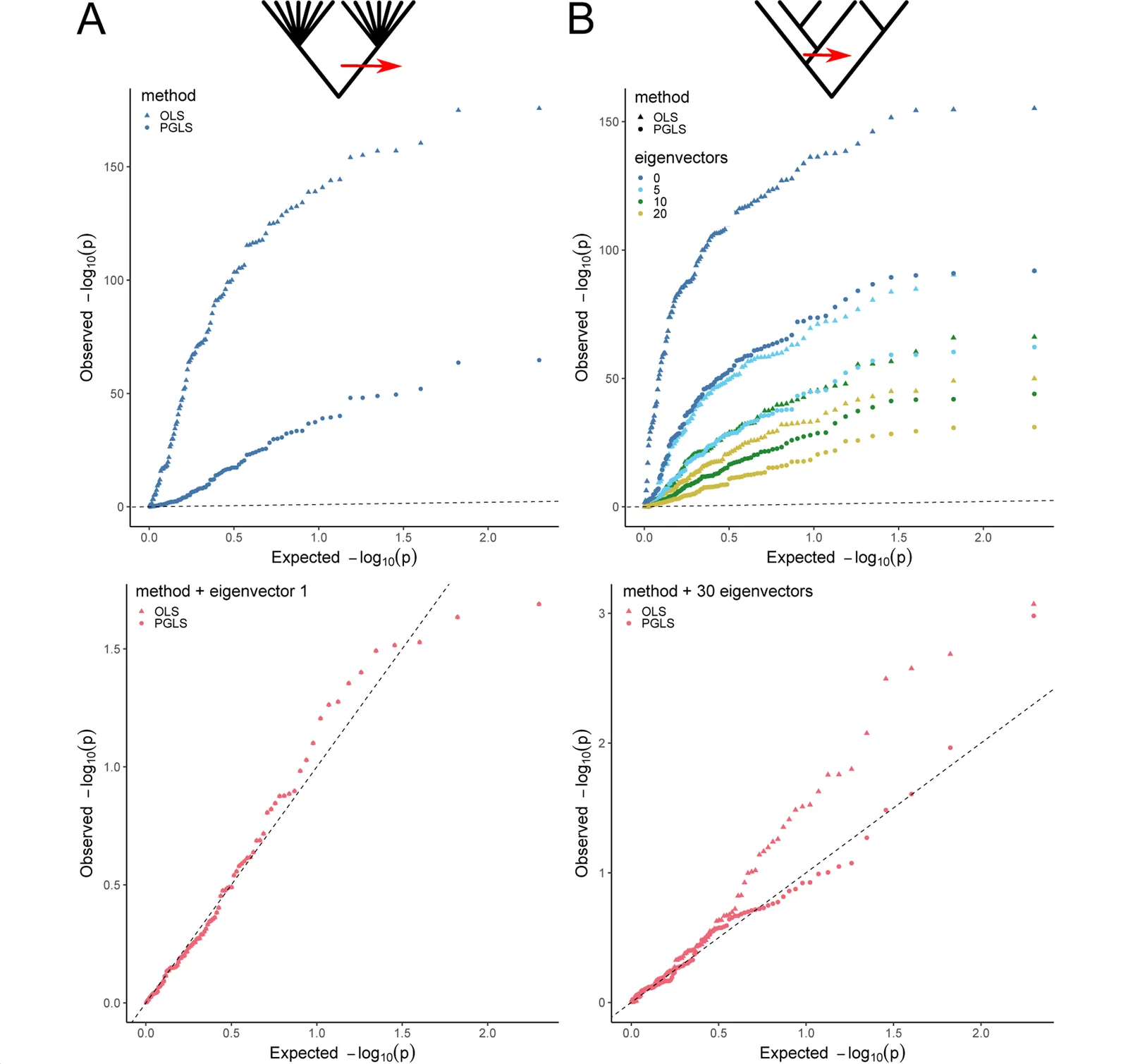
Quantile-quantile plots (QQ plots) showing the performance of different methods in controlling phylogenetic confounding. (A) QQ plots showing the performance of different methods in controlling confounding in Felsenstein’s worse case (FWC) with a non-Brownian shift in both predictor and outcome variables. For simplicity, only 7 tips are shown for each clade; in simulations, each clade contains 100 tips. The red arrow represents the non-Brownian shift on the branch giving rise to one of the two clades. The dotted line shows the case when the observed *p*-values exactly match the expected null *p*-values. The top panel shows the case when the methods fail to control the confounding, and the bottom panel shows the case when the methods manage to control the confounding. The dots for OLS + eigenvector $v_{1}$ and $\text{PGLS}+v_{1}$ are completely overlapping. (B) QQ plots showing the performance of different methods in controlling confounding in model related by a Yule tree with a non-Brownian shift in both predictor and outcome variables. For simplicity, only 6 tips are shown; in simulations, the Yule tree contains 100 tips. The red arrow represents the non-Brownian shift on one of the branches. The dotted line shows the case when the observed *p*-values exactly match the expected null *p*-values. The top panel shows the case when the methods fail to control the confounding, and the bottom panel shows the case when the methods manage to control the confounding.

For FWC with one shift on one of the clades, we ran a total of 100 replicates and created QQ plots for four methods: OLS, OLS with eigenvector *v*_1_, PGLS, and PGLS with $v_{1}$. (Fig. 3A) We applied the same tool to a slightly more complicated scenario, where there is a non-Brownian shift on a branch of a Yule tree (Fig. 3B). We generated a Yule tree of 100 species and introduced a shift on one of the branches. We simulated x and y as arising from independent Brownian motions along the tree, and we generated the shift by adding a normal random variable with a large standard deviation (s=100) to the species that are descendants of the branch where the shift occurs. We ran a total of 100 replicates and we created QQ plots for OLS and PGLS with different numbers of eigenvectors (from 0 up to 30).

**Fig 4:**
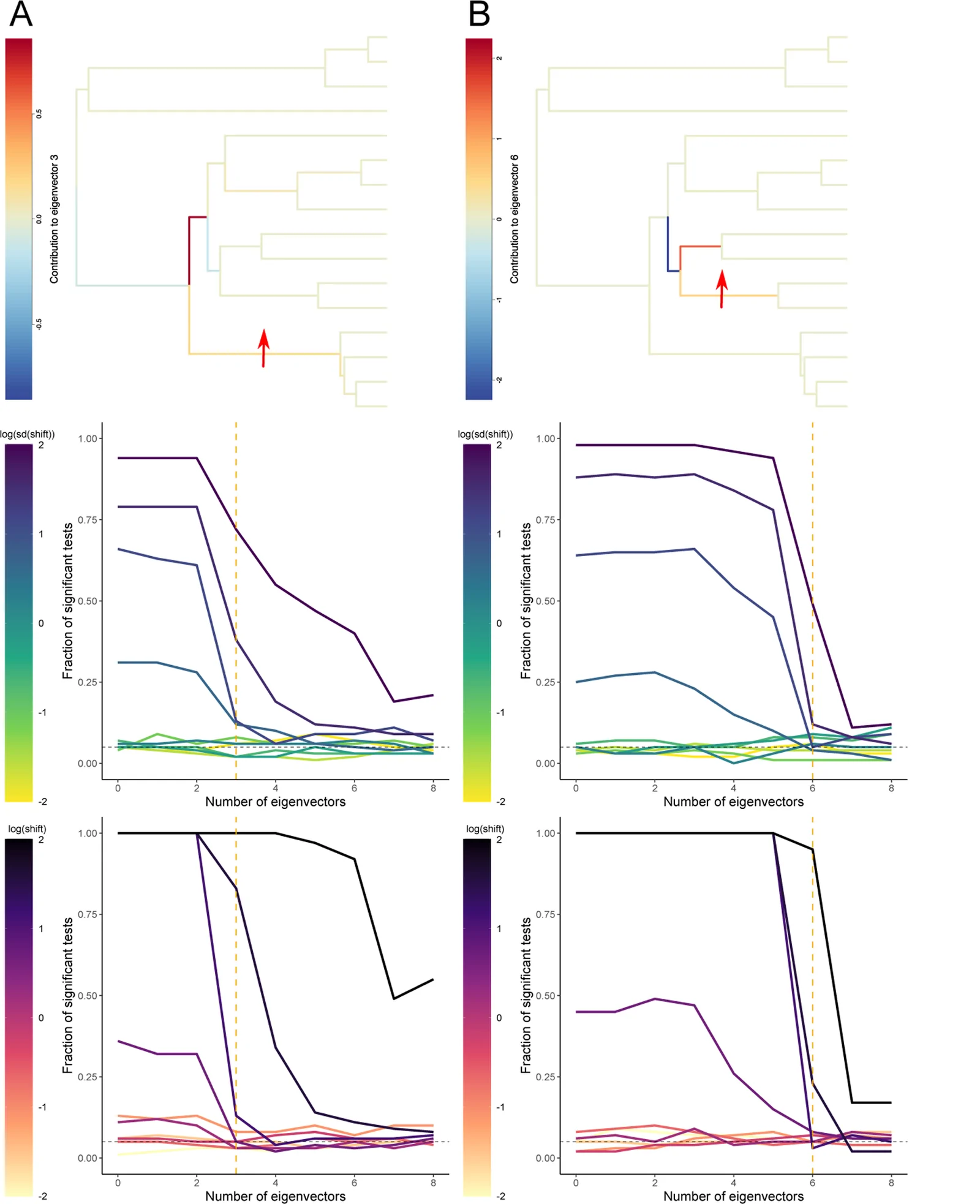
The performance of using eigenvectors in phylogenetic generalized least squares (PGLS) to remove phylogenetic confounding in a model with 16 tips related by a Yule tree with non-Brownian shifts in both predictor and outcome variables. The top panels show the contribution of branches to one eigenvector of the variance-covariance matrix C. The red arrows indicate the branches where the shifts occur. The middle panels show the performance of using eigenvectors in PGLS to remove phylogenetic confounding in the model, where the shift is simulated by adding a normal random variable with varying standard deviation to the species that are descendants of the branch. The horizontal axis shows the number of eigenvectors included as covariates, and the vertical axis shows the fraction of tests that would be significant at the 0.05 level. The vertical orange dashed lines show the number of eigenvectors corresponding to the dimension in the top panels. The bottom panels show the performance of using eigenvectors in PGLS to remove phylogenetic confounding in the model, where the shift is simulated by adding a constant of varying size to the species that are descendants of the branch. The horizontal axis shows the number of eigenvectors included as covariates, and the vertical axis shows the fraction of tests that would be significant at the 0.05 level. The vertical orange dashed lines show the number of eigenvectors corresponding to the dimension in the top and middle panels. (A) The shift falls on a branch that contributes the most to $v_{3}$ of C among all the first 8 eigenvectors. (B) The shift falls on a branch that contributes the most to $v_{6}$ of C among all the first 8 eigenvectors.

Building on the last scenario, we are interested in how many eigenvectors we need to include as covariates in the model to mitigate the confounding when there is a non-Brownian shift of different sizes on different branches of the tree. We generated a Yule tree with 16 tips, and we simulated x and y arising independently from a Brownian motion along each of the trees. Then we selected a branch where the shift occurs, and we considered two different types of shifts. The first type is the same as in the previous simulations, where the shift is generated by adding a normal random variable with a varying standard deviation to x and y values of the species that are descendants of the branch where the shift occurs. Biologically, this can be interpreted as an acceleration of the evolutionary rate on that particular branch where the shift occurs (*sensu* Landis and Schraiber, 2017). The second type of shift is generated by adding a constant of a varying size to x and y values of the species that are descendants of the branch where the shift occurs. A shift of this type can be interpreted as a shift in the optimal trait value within the corresponding clade. As in the previous simulations with no shifts, we compared the performance of using different numbers of eigenvectors in OLS and PGLS for phylogenetic regression. For each combination of shift position, shift type, and shift size, we ran a total of 100 replicates (Fig. 4).

### Application to gene expression data from cichlids

**Fig 5:**
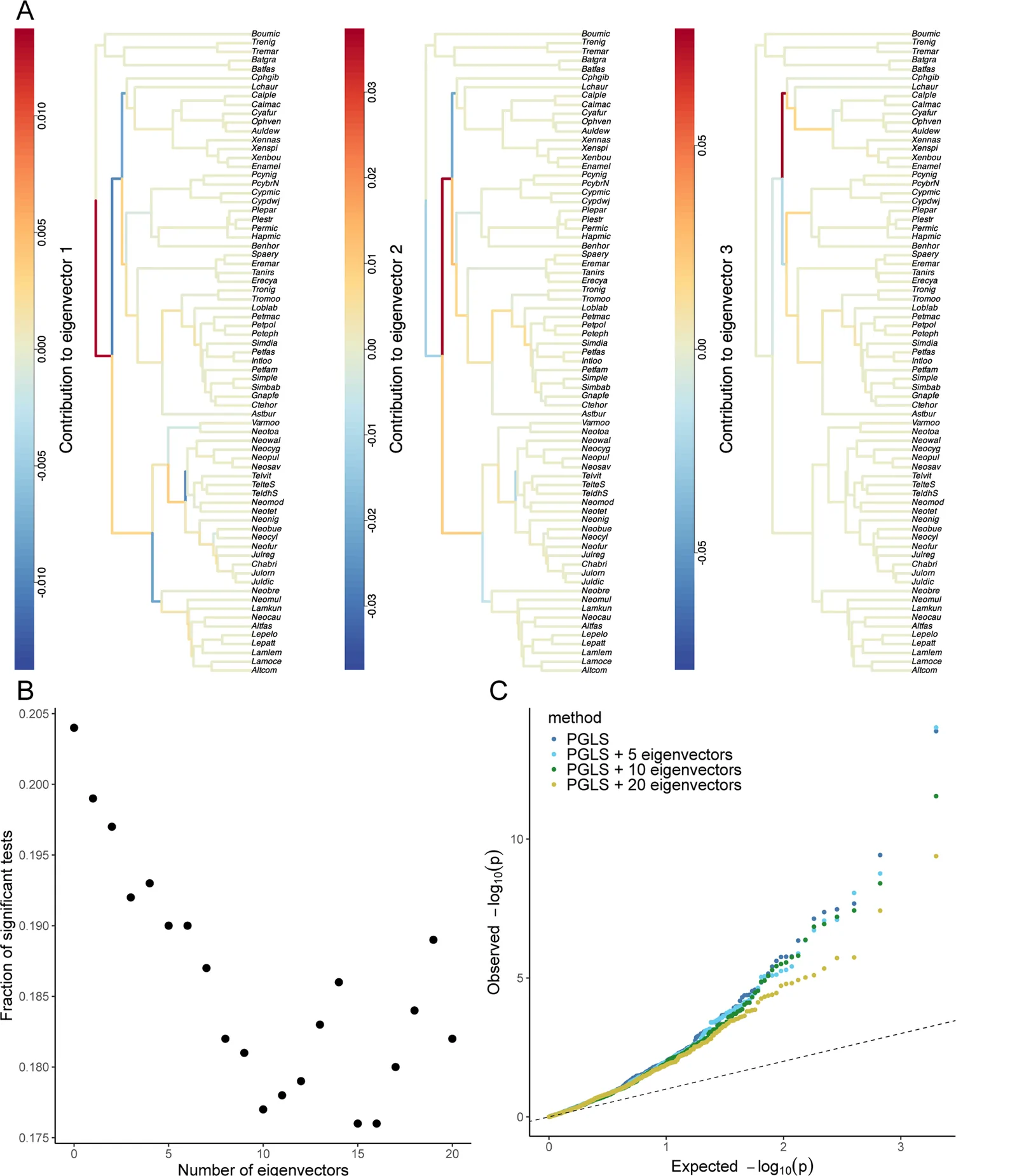
Effect of including phylogenetic eigenvectors on detection of coevolution of gene-expression levels in African cichlids. (A) Contributions of the branches to the first 3 eigenvectors of the phylogenetic variance-covariance matrix C of the phylogenetic tree for the 73 cichlid species. (B) The overall number of significant pairs first decreases and then fluctuates as more eigenvectors are included in PGLS. The horizontal axis indicates the number of eigenvectors included as fixed effects, and the vertical axis shows the proportion of significant pairs out of 1000 randomly selected pairs. (C) QQ plot showing the performance of using different numbers of eigenvectors included as fixed effects in PGLS. The dotted line shows the case when the observed *p*-values exactly match the expected null *p*-values.

As an illustration, we used data on gene expression from across a radiation of African rift lake cichlids from El Taher et al. (2021). Though not the original purpose of the study, we followed the study design of Cope et al. (2020) in testing whether pairs of genes showed evidence of co-evolution in their mRNA counts within the same tissue. Using the approach described above, we visualized how the first three eigenvectors capture the phylogenetic structure of the 73 species involved (Fig. 5A). We then selected 1000 random pairs of protein-coding genes that have non-zero gene expressions in lower pharyngeal jaw bones across all the species, and we performed PGLS with eigenvectors to see how some correlations can be spurious due to uncorrected phylogenetic confounding (Fig. 5B). Finally, we created QQ plots for the tests on these 1000 pairs with varying numbers of eigenvectors included as fixed effects (Fig. 5C).

## Results

### Description of simulation results

To examine the visualization tool we developed, we first checked how the branches contribute to the first eigenvector $v_{1}$ of $\overset{⌣}{C}$ in FWC. Since the only phylogenetic structure in this scenario is the split between the two clades, we expect $v_{1}$ to capture this split. This is exactly what we observe in Fig. 1B, as the two branches from the root splitting the two clades contributes the most to $v_{1}$, while all the other branches have nearly no contributions.

We also looked at the contributions of branches to the first three eigenvectors of $\overset{⌣}{C}$ on a Yule tree of 10 species. As shown in Fig. 1C, $v_{1}$ captures the largest split between the two largest clades as well as some splits within these clades. $v_{2}$ captures the split between the two-species clade and three-species clade within the large six-species clade, finer and more recent phylogenetic structure than in $v_{1}$. $v_{3}$ explains the split of the outgroup from the rest of the five species in the large 6-species clade. More generally, the dimension of the largest variance among all the species would generally correspond to the splits or branches that give rise to the most even partition of the species. The visualization method can also be easily extended to realizations of Σ matrix other than $\overset{⌣}{C}$ (see Figs. S1, S2, and S3). In Section 5 of the Supplementary Material, we explore the impact of centering on our visualization of the eigenvectors.

Fig. 2 shows the false positive rate after adding eigenvectors in OLS and PGLS for different types of trees when the data is generated by a pure Brownian motion with no additional shifts. In general, we see that using eigenvectors in conjunction with a random effects term capturing the phylogenetic structure (i.e., with PGLS) performs much better than using eigenvectors without the random effects term (i.e., with OLS); recall that using eigenvectors with OLS is essentially identical to the Phylogenetic Eigenvector Regression approach developed by Diniz-Filho et al. (1998), which is known to underperform PGLS under a purely Brownian model (Rohlf, 2001). As we have discussed, including eigenvectors of C as fixed effects can correct for punctate effects that are specific to a branch or region of the tree, whereas including C as the covariance structure in PGLS does a better job in correcting for phylogenetic confounding that is diffuse across the tree. In our simulations, the trees generally have complex structure, and thus including the eigenvectors in OLS ignores some of the later dimensions and fails to correct for diffuse confounding that occurs due to recent splits and other more subtle features of the phylogeny. We also generally observe that for OLS, adding more eigenvectors decreases the false positive rates, while adding more eigenvectors has little effect on the false positive rates when using PGLS. As later eigenvectors capture finer-scale structure of the phylogeny, including more eigenvectors in OLS can indeed correct for more of the smaller phylogenetic confounding. In contrast, in PGLS, the diffuse structure of the phylogeny is already taken into account by including C in the random effects term, and thus additional eigenvectors are redundant. Another clear pattern is that for larger trees (with more species), the false positive rate of using the same number of eigenvectors in OLS also tends to be larger, meaning that one would need to include more eigenvectors in OLS to correct for phylogenetic confounding. Intuitively, this is because the same number of eigenvectors capture a smaller proportion of the tree in a larger tree compared to a smaller tree. Analyses on non-ultrametric trees also generated similar results (Fig. S6).

Among the four types of trees we simulated, caterpillar trees stand out as the false positive rates of using eigenvectors in OLS drop much more quickly compared to other types of trees (Fig. 2B). This is because caterpillar trees have relatively simple structures, and when the internal branches are short, part of the caterpillar trees can be approximated as a star tree, thus collapsing into something similar to what we observe in FWC: even though there are multiple species, there is very little structure and thus can be captured by the first few eigenvectors. Interestingly, for OLS, after having 5 to 6 eigenvectors, adding more eigenvectors does not further decrease the false positive rate by much, and it remains an open question how the false positive rate curve would behave as the number of eigenvectors included as fixed effects approaches the number of species in the tree — when too many eigenvectors are included, the OLS model simply cannot be estimated because there are too many parameters to estimate compared with the number of observations.

Although false positive rates provide us with a summary of the results of multiple replicate tests, we also lose information by collapsing a list of values into one. Here we show how QQ plots of *p*-values can help us judge whether the confounding effect of phylogenetic structure is plausibly controlled. Expanding on the simulations of FWC with one shift in Schraiber et al. (2024), we created QQ plots for 100 replicates on a tree composed of two clades, each containing 100 species. As the first eigenvector $v_{1}$ can capture the split of the two clades, we expected both OLS and PGLS with the first eigenvector to be able to remove the phylogenetic confounding. This is exactly what we observe in Fig. 3A, where both methods that include $v_{1}$ have the observed *p*-values aligning with the expected *p*-values (bottom panel), whereas both methods that do not include $v_{1}$ as fixed effect give rise to a large number of much smaller *p*-values than expected (top panel). It is also noteworthy that when including $v_{1}$ as covariate, the results of OLS and PGLS overlap exactly, which agrees with the results from Schraiber et al. (2024). We can mathematically explain this result by applying equations (16) and (18) in Schraiber et al. (2024) to FWC (see Section 8 of the supplementary material).

We also created QQ plots for cases where there is a shift on the 100-tip Yule tree (Fig. 3B). Similar to previous simulations, the group of methods using PGLS in general perform better than their OLS counterparts, and for OLS, as more eigenvectors are included, the observed distribution of *p*-values get closer to that expected under the null hypothesis. However, because there is now a non-Brownian shift in the data, we see that additional eigenvectors helps to mitigate the confounding. As the top panel shows, even 20 eigenvectors fail to fully correct the confounding in the phylogenetic regression; the bottom panel shows that we need to include as many as 30 eigenvectors in combination with PGLS to control the false positive rate. On the other hand, even with 30 eigenvectors, OLS fails to generate uniform *p*-values. This illustrates that including eigenvectors as covariates complements PGLS. We have a Yule tree with lots of diffuse phylogenetic structure, so we need PGLS to correct for the overall confounding. On the other hand, we also have a large shift on one of the branches, which leads to confounding that’s out of scale with the tree and requires eigenvectors as covariates.

However, from the previous case it is not immediately clear why we would need 30 eigenvectors to fully correct for the confounding caused by the shift. Therefore, we turned to a much smaller tree containing just 16 species and investigated how the size of the shift and where it occurs on the tree impact the number of eigenvectors we need to include in phylogenetic regressions to fully mitigate the confounding. We considered two types of shifts: one corresponding to an acceleration of the evolutionary rate on the branch, and the other corresponding to a change in the optimal trait value for the clade. We focus our discussion on two representative scenarios (for more scenarios, see Figs. S7, S8, and S9). In Fig. 4A, the shift occurs on the branch that leads to the four-species clade at the bottom of the tree, whereas in Fig. 4B, the shift occurs on a more recent branch that gives rise to only two species. Across both scenarios and both types of shifts, a common pattern is that the false positive rate increases with the shift size but decreases with the number of eigenvectors included as fixed effects. What is more noteworthy, however, is that the false positive rate drops sharply in most cases once we include $v_{3}$ in Fig. 4A and $v_{6}$ in Fig. 4B. Using the tool introduced at the beginning to visualize the contributions of branches to the eigenvectors of the variance-covariance matrix, we see that among the first eight eigenvectors, the branch where the shift occurs in Fig. 4A contributes mostly to $v_{3}$, and in the case of Fig. 4B, the branch contributes mostly to $v_{6}$. This explains why including $v_{3}$ and $v_{6}$ in the two cases would be especially helpful, since these eigenvectors capture the branches where the shifts occur. Comparing the middle and bottom panels, which correspond to the two types of shifts we simulated, we observe that the results are qualitatively similar for both types of shifts. The combined strategy is slightly more vulnerable to cases when there is a large shift on the optimal trait value, since this type of shift was generated by adding a large constant to the species that are descendants of the branch where the shift occurs. The other type of shift, corresponding to an acceleration of evolutionary rate, was generated by adding a random normal variable with some large standard deviation, so across replicates, the effect of the shift is smaller on average.

In empirical data analyses, it is often unclear whether there is a non-Brownian shift on the phylogenetic tree, and if there is, where the shift occurs. Therefore, we revisited the same case above from an alternative perspective — how the false positive rate can be impacted by where the shift falls on the tree if no eigenvectors are included at all in PGLS (Fig. S10). We utilized the visualization tool for the contributions of branches to the eigenvectors of the variance-covariance matrix and computed the overall contributions of the branches (see Section 6 of the supplementary material), which we used to rank the branches. In general, when the shift falls on a branch that has a higher ranking (i.e., one that makes a large overall contribution), the false positive rate of PGLS alone is higher. This implies that the branches making the most overall contributions are most likely to create large confounding in phylogenetic regressions. However, branches that make less overall contributions can also create large confounding when the shift size is large.

We further looked at cases with multiple shifts on the tree (Figs. S11, S12, and S13). In general, we observe the same pattern as in the case of a single shift on the tree: if we include all the leading eigenvectors as fixed effects, then the minimum number of eigenvectors we need to include is equal to the last dimension to which the branches where the shifts occur make large contributions.

Finally, we investigated cases when β, the true effect of x on y is non-zero (Figs. S15, S16, and S17). In general, our combined method tends to lose power if too many eigenvectors are included as fixed effects. This is especially true when the tree size and the effect size β are small (Fig. S15). When the tree size is small, as is in cases of sparse sampling, it is expected that tests will have lower power; when the effect size β is small, the value would be close to zero and hard to detect unless more data (number of species) are included. We then looked at cases when there is also a shift on the tree, and our combined method has the highest power when the shift size and β are large, corresponding to cases that deviate most from the null scenario (Figs. S16 and S17).

### Empirical example

We first applied our visualization tool to see how the eigenvectors capture the structure of the phylogenetic tree of the 73 species of African rift lake cichlid fishes (Fig. 5A). Similar to our simulations, $v_{1}$ and $v_{2}$ capture the splits between large and medium clades, whereas *v*_3_ captures the structure on finer structures and more recent splits.

We then ran PGLS with varying numbers of eigenvectors to test the number of pairs of genes that have significant correlations. As is shown in Fig. 5B, when no eigenvectors are included as fixed effects, around 20% of the 1000 randomly selected pairs show significant correlations. As we include more eigenvectors, this fraction keeps decreasing until around 10 eigenvectors. When even more eigenvectors are included, the fraction of significant tests starts to fluctuate between 17.5% and 19%. The initial drop in the fraction of significant tests suggests that some of the correlations can be spurious if we fail to use the first few eigenvectors to remove the large confounding effect of the tree. The later fluctuation of the fraction suggests that adding more eigenvectors might not be necessarily better; after correcting for the large confounding effects using the first few eigenvectors, adding more eigenvectors might do little to remove additional confounding and will reduce power.

Finally, we created a QQ plot for the tests involving these 1000 pairs of genes (Fig. 5C). A key difference between the QQ plot here and the QQ plots in Fig. 3 is that in Fig. 3, we know that x and y evolve independently, and thus we know the true distribution of *p*-values, whereas here we do not know in reality how many of the 1000 randomly selected pairs indeed have significant correlations. In a statistical genetics context, it has been argued that if most traits are highly polygenic (or even omnigenic, Boyle et al., 2017), then we expect a general inflation of *p*-values (Yang et al., 2011). Therefore, we can only use the null distribution, a uniform distribution, as expected *p*-values, to test whether there are more significant pairs than the 5% threshold. As Fig. 5C shows, there are indeed quite a lot of pairs that have significant correlations across all the methods, but adding more eigenvectors does help us remove at least part of the phylogenetic confounding and reduce false positive rates.

## Discussion

In this paper, we have advanced the argument that, under some biologically realistic scenarios, a mixed strategy of including eigenvectors of the phylogenetic variance-covariance matrix as fixed effects and using the same matrix to model correlated residuals/random effects performs better than either approach on its own. This may be a counterintuitive proposal. These approaches were previously seen as competing alternatives when they were first developed in phylogenetic comparative methods (Rohlf, 2001; Freckleton et al., 2011). And at first glance, it seems like such a mixed strategy is essentially “double-counting” the phylogeny as it appears twice in the regression equation. But as we argue here and elsewhere (Schraiber et al., 2024), comparative biologists can profit from having seen the evolution of parallel developments in statistical genetics. Statistical geneticists re-discovered the use of principal components to control for ancestry (Zhang et al., 2003; Price et al., 2006), and then subsequently re-discovered the idea of using a LMM for the same purpose (Yu et al., 2006; Kang et al., 2008) — although in the latter case, both comparative biologists and statistical geneticists re-invented an approach developed by quantitative geneticists decades earlier (Henderson, 1984). Statistical geneticists subsequently had the insight that these two could be combined, which to our knowledge had not been considered in comparative biology. The mixed strategy appeared to work so well in that context for much the same reasons we have documented here: disproportionally large effects are not captured well by standard LMM/PGLS approaches and that including the eigenvectors can therefore rescue the statistical model (Price et al., 2006; Tucker et al., 2014) — this was demonstrated mathematically by Schraiber et al. (2024). We emphasize that our results also are in agreement with previous assessments of the use of PVR (Rohlf, 2001; Laurin, 2010; Freckleton et al., 2011; Adams and Church, 2011; Chen and Niu, 2024); except in corner cases such as FWC, *only* including eigenvectors in an OLS regression is a suboptimal strategy. In that case, as our visualization tool shows, the two branches from the root make the largest contribution to the eigenvectors with non-zero eigenvalues and hence also the most overall contributions.

Much of the debate surrounding the PVR approach, both by supporters and critics, was on how many eigenvectors to include in the analysis (Adams and Church, 2011; Freckleton et al., 2011; Diniz-Filho et al., 2012a,b). If we do not know where on the tree shifts have occurred, it remains an open problem as to know in advance how many eigenvectors to include; but we emphasize that this is still very much an open problem in statistical genetics as well. Adding eigenvectors may mitigate confounding but will come at a loss of power. Here we point out that if one is doing many phylogenetic tests, for example if one is using comparative data to find associations between traits and genomic features (often referred to as ‘PhyloG2P’ tests Smith et al., 2020), QQ plots are one way to assess whether the confounding has been removed but this is not a sure fire guide as the null distribution of *p*-values will depend on the genetic architecture of the traits (Yang et al., 2011). Nonetheless statistical geneticists have gained rough intuition of what might be an appropriate number in a given scenario and we see no reason why phylogenetic biologists would not do the same. Last, we emphasize that the approaches presented here can be used in comparative studies even if one does not include eigenvectors in the model. For example, one could map the contribution of different branches to the leading eigenvectors and either include that branch as an indicator variable (following the suggestion of Uyeda et al., 2018) or else assess whether one’s analysis is potentially susceptible to being misled by unobserved shifts on these key branches.

Although phylogenetic biologists can certainly learn a lot from statistical geneticists, we end by emphasizing that this is a two-way street. Indeed, as we mentioned above, it is now apparent that there was a missed opportunity for statistical geneticists to take inspiration from PVR and PGLS from phylogenetics rather than re-inventing it themselves years later (for a recent example of a human genetics method that draws inspiration from PGLS, see Patel et al., 2025). The visualization technique we have developed here, which extends theoretical developments in statistical genetics (Fan et al., 2022), could provide new insights into how exactly population structure confounds trait-mapping and related problems and how to mitigate its effects. Looking forward, as tree-thinking becomes increasingly central to the theory and practice of statistical and population genetics (Lewanski et al., 2024; Nielsen et al., 2025), we anticipate that methods developed in phylogenetic comparative biology will become even more pertinent to these fields.

## Supplementary Material

Supplement 1

## Data Availability

Code used to generate the results in this paper can be found at https://github.com/applied-phylo-lab/Phylo_PC.
