# Supplementary material for: Characterizing and mitigating confounding by unreplicated evolutionary events in phylogenetic regression
Source: bioRxiv. 2025 Dec 5:2025.12.04.692436. Preprint. [Version 1] doi: 10.64898/2025.12.04.692436 (PMC12822681; doi:10.64898/2025.12.04.692436)
Supplement: Supplementary file 1 [file NIHPP2025.12.04.692436v1-supplement-1.pdf]

bioRxiv preprint doi: https://doi.org/10.64898/2025.12.04.692436; this version posted December 5, 2025. The copyright holder for this preprint (which was not certified by peer review) is the author/funder, who has granted bioRxiv a license to display the preprint in perpetuity. It is made available under a CC-BY 4.0 International license.

Supplementary material for: Characterizing and mitigating confounding by unreplicated evolutionary events in phylogenetic regression

Qifan Wang[1], Michael D. Edge[2], Joshua G. Schraiber[2,*] & Matt Pennell[1,*]

[1]*Department of Computational Biology, Cornell University*
[2]*Department of Quantitative and Computational Biology, University of Southern California*
[*]Corresponding authors: schraibe@usc.edu, mpennell@cornell.edu

Contents

1 Contributions of each branch to the eigenvectors of eGRM **2**

2 Relationship between eGRM and variance covariance matrix **3**

3 Relationship between eigenvectors of distance matrix and variance-covariance matrix **6**

4 Eigenvectors of matrices for non-ultrametric trees **8**

5 Notes on using non-centered variance-covariance matrix **10**

6 Overall contributions of each branch **13**

7 Connection between PGLS and PGLMM **14**

8 Performance of different methods in Felsenstein's Worst Case **14**

9 Supplementary Methods **16**

10 Supplementary Figures **19**

bioRxiv preprint doi: https://doi.org/10.64898/2025.12.04.692436; this version posted December 5, 2025. The copyright holder for this preprint (which was not certified by peer review) is the author/funder, who has granted bioRxiv a license to display the preprint in perpetuity. It is made available under a CC-BY 4.0 International license.

1 Contributions of each branch to the eigenvectors of eGRM

In statistical geneticists, the equivalent of the phylogenetic variance-covariance matrix C would be the genetic relatedness matrix [GRM, see 9] or its expected version [sometimes called an eGRM, see 3], both of which are used to capture genetic relatedness between individuals. The data used to generate these two matrices are usually some $n \times m$ genotype matrix G, where each of the n rows represents an individual, and each of the m columns represents a site (or a SNP). The calculation of GRM is directly based on G, whereas the calculation of eGRM is usually based on some tree or ARG (ancestral recombination graph) inferred from G. Both GRM and eGRM are collapsed over the number of sites and have a dimension of $n \times n$. Below we briefly show the key steps of calculating an eGRM from a tree $\mathcal{T}$ that is inferred from G.

Let $x(e)$ be the binary vector for a branch $e \in \mathcal{T}$, where $x(e)[i]$ is 1 if the i-th tip is a descendant of branch e and 0 otherwise. Let $l(e)$ be the length of branch e. Then we can get the eGRM of the branch e:

$$\Sigma(e) = \frac{\left(x(e) - \overline{x(e)}\mathbf{1}\right)\left(x(e) - \overline{x(e)}\mathbf{1}\right)^T}{\overline{x(e)}\left(1 - \overline{x(e)}\right)}. \tag{1}$$

The eGRM of the entire tree would be a weighted sum of branch eGRMs:

$$\Sigma(\mathcal{T}) = \sum_{e \in \mathcal{T}} \Sigma(e) \frac{l(e)}{\sum_{e' \in \mathcal{T}} l(e')} \tag{2}$$

$$= \sum_{e \in \mathcal{T}} \tilde{\Sigma}(e), \tag{3}$$

where $\tilde{\Sigma}(e)$ is a weighted eGRM representing the contribution of branch e to the tree eGRM.

Now we show the approach to calculate the contributions of each branch to the eigenvectors of eGRM. Same as using C or $\breve{C}$, we first perform an eigenvector decomposition of the tree eGRM:

$$\begin{aligned}\Sigma(\mathcal{T}) &= V \Lambda V^T \\ &= \sum_d \lambda_d v_d v_d^T.\end{aligned}$$

Then we can perform a linear regression:

$$v_d v_d^T = \sum_{e \in \mathcal{T}} w_d(e) \tilde{\Sigma}(e).$$

Since for large matrices there is no analytical solution for eigenvectors, it is impossible to mathematically derive $w_d(e)$ in a closed form. Therefore, in practice, the only way to calculate the contributions of each branch to the eigenvectors of a general eGRM would be to perform a linear regression without an intercept.

bioRxiv preprint doi: https://doi.org/10.64898/2025.12.04.692436; this version posted December 5, 2025. The copyright holder for this preprint (which was not certified by peer review) is the author/funder, who has granted bioRxiv a license to display the preprint in perpetuity. It is made available under a CC-BY 4.0 International license.

2 Relationship between eGRM and variance covariance matrix

From equations (1) to (3), we know that:

$$\begin{aligned}\tilde{\Sigma}(e) &= \frac{\left(x(e) - \overline{x(e)}\mathbf{1}\right)\left(x(e) - \overline{x(e)}\mathbf{1}\right)^T}{\overline{x(e)}\left(1 - \overline{x(e)}\right)} \frac{l(e)}{\sum_{e' \in \mathcal{T}} l(e')} \\ &= H \frac{x(e)x(e)^T}{\overline{x(e)}\left(1 - \overline{x(e)}\right)} H \frac{l(e)}{\sum_{e' \in \mathcal{T}} l(e')}, \end{aligned} \quad (4)$$

with

$$H = I - \frac{1}{n}\mathbf{1}\mathbf{1}^T,$$

where I is the identity matrix and n is the number of tips of the tree $\mathcal{T}$.

Traditionally, phylogeneticists use a variance-covariance matrix (VCV or C) by assuming that traits evolve following a Brownian motion across the tree. As variance-covariance matrix explicitly models the evolution of traits, it is natural to consider using variance-covariance matrix to compute eigenvectors for covariates in phylogenetic regression, which is shown in our previous paper [7]. Similar to eGRM, here we propose a branch version of a variance-covariance matrix:

$$C(e) = \sigma^2 l(e) x(e) x(e)^T. \quad (5)$$

The intuition is that if tips i and j are both descendants of a branch e, then the branch would contribute $\sigma^2 l(e)$ to their covariance under a Brownian motion, and thus we would have:

$$C(e)[i,j] = \begin{cases} \sigma^2 l(e) & \text{if tips } i \text{ and } j \text{ are descendants of branch } e, \\ 0 & \text{otheriwse .} \end{cases}$$

Note that this equation also works when i and j happen to be the same individual. The variance-covariance matrix for the whole tree $\mathcal{T}$ would then be the sum of variance-covariance matrices for the branches:

$$C(\mathcal{T}) = \sum_{e \in \mathcal{T}} C(e). \quad (6)$$

Combining equations (4) and (5), we can show:

$$\tilde{\Sigma}(e) \propto H \frac{C(e)}{\overline{x(e)}\left(1 - \overline{x(e)}\right)} H.$$

bioRxiv preprint doi: https://doi.org/10.64898/2025.12.04.692436; this version posted December 5, 2025. The copyright holder for this preprint (which was not certified by peer review) is the author/funder, who has granted bioRxiv a license to display the preprint in perpetuity. It is made available under a CC-BY 4.0 International license.

From the equation above, we observe that the differences between variance-covariance matrix and (weighted) eGRM of a branch are two-fold. First, the variance-covariance matrix does not mean-center the data, while eGRM is mean-centered both row- and column-wise. Second, variance-covariance matrix does not normalize the data whereas eGRM does.

We argue that mean-centering the matrix is an important step for including eigenvectors into phylogenetic regressions. In Principal Component Analysis (PCA), one would mean-center the data before calculating the variance-covariance matrix and performing the eigenvector decomposition; otherwise, the data could be biased by the mean. As eGRM is obtained after mean-centering the data $x(e)$, performing an eigenvector decomposition on eGRM would be equivalent to performing PCA on the original data, except that the data are also normalized, such that the branches that give rise to more uneven partitions ($x(e)$ being mostly zeroes or ones) among the tip species are given more weights. Since the variance-covariance matrix is obtained without mean-centering the data first, directly performing an eigenvector decomposition can generate biased PCs. Here we show how eigenvector decomposition can change before and after double-centering.

Suppose that the eigenvector decomposition of the variance-covariance matrix is:

$$C(\mathcal{T}) = V\Lambda V^T.$$

Then the doubled-centered variance-covariance matrix would be:

$$\begin{aligned}\breve{C}(\mathcal{T}) &= HC(\mathcal{T})H \\ &= HV\Lambda V^T H \\ &= (HV)\,\Lambda\,(HV)^T.\end{aligned}$$

Note that this is not an eigenvector decomposition of $\breve{C}(\mathcal{T})$, because the columns of HV are not orthonormal. Now the i-th column of the matrix would be:

$$\left(I - \frac{1}{n}\mathbf{1}\mathbf{1}^T\right) v_i = v_i - \overline{v_i}\mathbf{1}.$$

And thus we have:

$$\begin{aligned}(v_i - \overline{v_i}\mathbf{1})^T (v_j - \overline{v_j}\mathbf{1}) &= v_i^T v_j - \overline{v_i}\mathbf{1}^T v_j - \overline{v_j} v_i^T \mathbf{1} + \overline{v_i}\overline{v_j}\mathbf{1}^T\mathbf{1} \\ &= v_i^T v_j - N\overline{v_i}\overline{v_j} \\ &= -N\overline{v_i}\overline{v_j}, \\ (v_i - \overline{v_i}\mathbf{1})^T (v_i - \overline{v_i}\mathbf{1}) &= v_i^T v_i - N\overline{v_i}^2 \\ &= 1 - N\overline{v_i}^2.\end{aligned}$$

While the above shows that the eigenvector decomposition of $\breve{C}(\mathcal{T})$ cannot be obtained via a simple

bioRxiv preprint doi: https://doi.org/10.64898/2025.12.04.692436; this version posted December 5, 2025. The copyright holder for this preprint (which was not certified by peer review) is the author/funder, who has granted bioRxiv a license to display the preprint in perpetuity. It is made available under a CC-BY 4.0 International license.

transformation from the $C(\mathcal{T})$, there is actually no analytical relationship between the eigenvector decomposition of $\breve{C}(\mathcal{T})$ and that of $C(\mathcal{T})$.

McVean [6] has suggested using $E(M)$ as the matrix for eigenvector decomposition, with:

$$E(M_{ij}) = \frac{1}{\overline{T}} \left(\frac{1}{n} \sum_{k=1}^{n} \overline{t_{ik}} + \frac{1}{n} \sum_{l=1}^{n} \overline{t_{lj}} - \frac{1}{n^2} \sum_{l=1}^{n} \sum_{k=1}^{n} \overline{t_{lk}} - \overline{t_{ij}} \right), \tag{7}$$

where $\overline{t_{ij}}$ is the expected coalescence time for tips i and j, and $\overline{T}$ is the expected total branch length in the tree. Note that McVean's $E(M)$ is derived from expected coalescence times, where the tree is not fixed. However, assuming that the tree is fixed, we eventually obtain the same matrix as the double-centered variance-covariance matrix. We have:

$$t_{ij} = T - \sum_{e \in \mathcal{T}} x(e)[i]x(e)[j]l(e). \tag{8}$$

The intuition is that the distance from the most recent common ancestor of tips i and j to the root of the tree would be the sum of the lengths of branches that have both i and j as descendants.

Plugging equation (8) into (7), we get:

$$\begin{aligned} E(M_{ij} \mid \mathcal{T}) = \frac{1}{T} \Bigg[& \frac{1}{n} \sum_{k=1}^{n} \left(T - \sum_{e \in \mathcal{T}} x(e)[i]x(e)[k]l(e) \right) \\ & + \frac{1}{n} \sum_{l=1}^{n} \left(T - \sum_{e \in \mathcal{T}} x(e)[l]x(e)[j]l(e) \right) \\ & - \frac{1}{n^2} \sum_{l=1}^{n} \sum_{k=1}^{n} \left(T - \sum_{e \in \mathcal{T}} x(e)[l]x(e)[k]l(e) \right) \\ & - \left(T - \sum_{e \in \mathcal{T}} x(e)[i]x(e)[j]l(e) \right) \Bigg]. \end{aligned}$$

Simplifying the above equation gives us:

$$\begin{aligned} E(M_{ij} \mid \mathcal{T}) &= \frac{1}{T} \sum_{e \in \mathcal{T}} l(e) \Bigg(x(e)[i]x(e)[j] - \frac{1}{n} \sum_{k=1}^{n} x(e)[i]x(e)[k] \\ &\quad - \frac{1}{n} \sum_{l=1}^{n} x(e)[l]x(e)[j] + \frac{1}{n^2} \sum_{l=1}^{n} \sum_{k=1}^{n} x(e)[l]x(e)[k] \Bigg) \\ &= \frac{1}{T} \sum_{e \in \mathcal{T}} l(e) \left(x(e)[i] - \overline{x(e)} \right) \left(x(e)[j] - \overline{x(e)} \right). \end{aligned} \tag{9}$$

bioRxiv preprint doi: https://doi.org/10.64898/2025.12.04.692436; this version posted December 5, 2025. The copyright holder for this preprint (which was not certified by peer review) is the author/funder, who has granted bioRxiv a license to display the preprint in perpetuity. It is made available under a CC-BY 4.0 International license.

We can rewrite equation (9) in matrix format:

$$\begin{aligned} E(M \mid \mathcal{T}) &= \frac{1}{T} \sum_{e \in \mathcal{T}} l(e) \left(x(e) - \overline{x(e)}\mathbf{1} \right) \left(x(e) - \overline{x(e)}\mathbf{1} \right)^T \\ &= \frac{1}{T} H \sum_{e \in \mathcal{T}} l(e) x(e) x(e)^T H \\ &\propto HC(\mathcal{T})H \\ &\propto \breve{C}(\mathcal{T}). \end{aligned}$$

Therefore, we have shown that McVean's $E(M)$ is proportional to $\breve{C}(\mathcal{T})$ if the tree $\mathcal{T}$ is fixed, and thus performing an eigenvector decomposition of $E(M \mid \mathcal{T})$ would be equivalent to performing the same analyses on $\breve{C}(\mathcal{T})$.

3 Relationship between eigenvectors of distance matrix and variance-covariance matrix

When the method of using eigenvectors in phylogenetic regressions (PVR) was first proposed, Diniz-Filho et al. [2] uses a double-centered distance matrix to get the eigenvectors. Here we show that this is equivalent to using a double-centered variance-covariance matrix.

The phylogenetic distance between tips i and j would be:

$$D[i, j] = 2t_{ij}. \tag{10}$$

We can also rewrite equation (8) in a matrix format:

$$t = T\mathbf{1}\mathbf{1}^T - \sum_{e \in \mathcal{T}} x(e) x(e)^T l(e). \tag{11}$$

Combining equations (10) and (11), and assuming a Brownian motion σ^2 of 1, we can derive the formula for the phylogenetic distance matrix of tree $\mathcal{T}$:

$$\begin{aligned} D(\mathcal{T}) &= 2 \left(T\mathbf{1}\mathbf{1}^T - \sum_{e \in \mathcal{T}} x(e) x(e)^T l(e) \right) \\ &= 2 \left(T\mathbf{1}\mathbf{1}^T - C(\mathcal{T}) \right). \end{aligned}$$

Now we show that if a vector v is an eigenvector of double-centered variance-covariance matrix $\breve{C}(\mathcal{T})$, then it is also an eigenvector of double-centered distance matrix $\breve{D}(\mathcal{T})$.

bioRxiv preprint doi: https://doi.org/10.64898/2025.12.04.692436; this version posted December 5, 2025. The copyright holder for this preprint (which was not certified by peer review) is the author/funder, who has granted bioRxiv a license to display the preprint in perpetuity. It is made available under a CC-BY 4.0 International license.

The fact that a vector v is an eigenvector of $\breve{C}\left(\mathcal{T}\right)$ means there exists some eigenvalue λ_1 such that:

$$HC(\mathcal{T})Hv = \lambda_1 v.$$

We need to show that there exists some eigenvalue λ_2 such that:

$$HD(\mathcal{T})Hv = \lambda_2 v.$$

That is:

$$2H\left(T\mathbf{1}\mathbf{1}^T - C(\mathcal{T})\right)Hv = \lambda_2 v.$$

We only need to show that there exists some eigenvalue λ_3 such that:

$$HT\mathbf{1}\mathbf{1}^T Hv = \lambda_3 v.$$

We note that the matrix on the left is a zero matrix because:

$$\begin{aligned} HT\mathbf{1}\mathbf{1}^T &= T\mathbf{1}\mathbf{1}^T - \frac{1}{n}\mathbf{1}\mathbf{1}^T T\mathbf{1}\mathbf{1}^T \\ &= T\mathbf{1}\mathbf{1}^T - \frac{T}{n}\mathbf{1}\left(\mathbf{1}^T\mathbf{1}\right)\mathbf{1}^T \\ &= T\mathbf{1}\mathbf{1}^T - T\mathbf{1}\mathbf{1}^T \\ &= \mathbf{0}. \end{aligned}$$

Therefore, v is an eigenvector for $HT\mathbf{1}\mathbf{1}^T H$ with corresponding eigenvalue $\lambda_3 = 0$, and thus v is also an eigenvector for $\breve{D}\left(\mathcal{T}\right)$ with corresponding eigenvalue $\lambda_2 = -2\lambda_1$. One thing to note here is that $\breve{C}\left(\mathcal{T}\right)$ and $\breve{D}\left(\mathcal{T}\right)$ would always differ in the signs of their eigenvalues. In fact, we can show that $\breve{C}\left(\mathcal{T}\right)$ would always generate non-negative eigenvalues, whereas $\breve{D}\left(\mathcal{T}\right)$ would always give non-negative eigenvalues.

To prove this, we can simply show that for any vector v:

$$v^T HC(\mathcal{T})Hv \geq 0.$$

Using the definition of variance-covariance matrix in equations (5) and (6), we have:

$$\begin{aligned} v^T HC(\mathcal{T})Hv &= v^T H \sum_{e\in\mathcal{T}} \sigma^2 l(e)x(e)x(e)^T Hv \\ &= \sum_{e\in\mathcal{T}} \sigma^2 l(e)\left(x(e)^T Hv\right)^2 \\ &\geq 0. \end{aligned}$$

bioRxiv preprint doi: https://doi.org/10.64898/2025.12.04.692436; this version posted December 5, 2025. The copyright holder for this preprint (which was not certified by peer review) is the author/funder, who has granted bioRxiv a license to display the preprint in perpetuity. It is made available under a CC-BY 4.0 International license.

4 Eigenvectors of matrices for non-ultrametric trees

In non-ultrametric trees, the variance-covariance matrix C of the trait for tree $\mathcal{T}$ can be computed following exactly the same equations (5) and (6), and after double-centering the variance-covariance matrix, the eigenvalues would still be all non-negative. However, when it comes to the distance matrix, since tips have different distances from the root of the tree, the equations in Section 3 cannot be applied. Here, we extend the discussion in Section 3 to non-ultrametric trees and show that after double-centering, the distance matrix and the variance-covariance matrix of the same tree still share the same eigenvectors.

First, we derive the distance matrix D of a tree $\mathcal{T}$. de Vienne et al. [1] have shown that the phylogenetic distance between two tips i and j of a tree can be expressed using the heights of the nodes (distances from nodes to the root of the tree). Let h_i, h_j, and $h_{MRCA[i,j]}$ be the heights of i, j, and their most recent common anestor, respectively. Then we have:

$$\begin{aligned} D[i,j] &= h_i + h_j - 2h_{MRCA[i,j]} \\ &= \sum_{e\in\mathcal{T}} x(e)[i]l(e) + \sum_{e\in\mathcal{T}} x(e)[j]l(e) - 2\sum_{e\in\mathcal{T}} x(e)[i]x(e)[j]l(e). \end{aligned} \tag{12}$$

We can rewrite equation (12) in a matrix format:

$$\begin{aligned} D(\mathcal{T}) &= \sum_{e\in\mathcal{T}} x(e)\mathbf{1}^T l(e) + \sum_{e\in\mathcal{T}} \mathbf{1}x(e)^T l(e) - 2\sum_{e\in\mathcal{T}} x(e)x(e)^T l(e) \\ &= \boldsymbol{h}\mathbf{1}^T + \mathbf{1}\boldsymbol{h}^T - 2C(\mathcal{T}), \end{aligned}$$

where the last line is obtained by assuming a variance of 1 for the Brownian motion and using $\boldsymbol{h} = \sum_{e\in\mathcal{T}} x(e)l(e)$ to denote the vector for heights of the tips.

Similar to Section 3, we can show that if a vector v is an eigenvector of $\breve{C}(\mathcal{T})$, then it is also an eigenvector of $\breve{D}(\mathcal{T})$. We only need to show that v is an eigenvector of double-centered matrix $H\left(\boldsymbol{h}\mathbf{1}^T + \mathbf{1}\boldsymbol{h}^T\right)H$. We note that:

$$\begin{aligned} H\boldsymbol{h}\mathbf{1}^T H &= \left(\boldsymbol{h}\mathbf{1}^T - \frac{1}{N}\mathbf{1}\mathbf{1}^T\boldsymbol{h}\mathbf{1}^T\right)H \\ &= \left(\boldsymbol{h}\mathbf{1}^T - \frac{\sum_i^N h_i}{N}\mathbf{1}\mathbf{1}^T\right)\left(I - \frac{1}{N}\mathbf{1}\mathbf{1}^T\right) \\ &= \boldsymbol{h}\mathbf{1}^T - \frac{1}{N}\boldsymbol{h}\mathbf{1}^T\mathbf{1}\mathbf{1}^T - \frac{\sum_i^N h_i}{N}\mathbf{1}\mathbf{1}^T + \frac{\sum_i^N h_i}{N^2}\mathbf{1}\mathbf{1}^T\mathbf{1}\mathbf{1}^T \\ &= \mathbf{0}. \end{aligned}$$

bioRxiv preprint doi: https://doi.org/10.64898/2025.12.04.692436; this version posted December 5, 2025. The copyright holder for this preprint (which was not certified by peer review) is the author/funder, who has granted bioRxiv a license to display the preprint in perpetuity. It is made available under a CC-BY 4.0 International license.

We also have:

$$H\mathbf{1}\boldsymbol{h}^T H = \left(H\boldsymbol{h}\mathbf{1}^T H\right)^T \\ = \mathbf{0}.$$

The calculations above give us:

$$\breve{D}(\mathcal{T}) = -2\breve{C}(\mathcal{T})$$

Therefore, we can show that v is an eigenvector of double-centered matrix $H\left(\boldsymbol{h}\mathbf{1}^T + \mathbf{1}\boldsymbol{h}^T\right)H$ with eigenvalue 0, and thus v is also an eigenvector of $\breve{D}\left(\mathcal{T}\right)$ with eigenvalue $-2\lambda_1$, where λ_1 is the corresponding eigenvalue for v for $\breve{C}\left(\mathcal{T}\right)$.

de Vienne et al. [1] have pointed out that for non-ultrametric phylogenetic trees, the distance matrix D can be non-Euclidean, and they suggest using matrix D^*, which is obtained by taking the element-wise square root of D. Since this is a non-linear transformation, we cannot mathematically show the relationship between eigenvectors of double-centered distance matrix $\breve{D}$ and double-centered element-wise square root distance matrix $\breve{D}^*$. However, by the definition of a Euclidean distance matrix D^*, one can represent the n tips in $\mathbb{R}^n$ space such that:

$$d\left(E_i, E_j\right) = D^*(\mathcal{T})[i,j],$$

where E_i and E_j are the embeddings of tips i and j. To compute the embeddings matrix E from the distance matrix D^*, we first square the matrix element-wise and then perform the double-centering transformation and multiply the matrix by a constant of $-\frac{1}{2}$. This gives us:

$$-\frac{1}{2}H\left[D^*(\mathcal{T})^2[i,j]\right]H = -\frac{1}{2}HD(\mathcal{T})H \\ = \breve{C}(\mathcal{T}).$$

Next, we perform eigenvector-decomposition of the double-centered variance-covariance matrix and the embeddings of the n tips can be expressed using the eigenvectors and eigenvalues:

$$\breve{C}(\mathcal{T}) = V\Lambda V^T, \\ E = V\Lambda^{\frac{1}{2}},$$

where E represents the embeddings of the n tips, with $E[i,d]$ representing the embedding of the i-th tip in the d-th dimension.

With the embeddings of n tips available, we can perform a PCA on the embeddings and use the projections onto the PC-axes to capture the phylogenetic structure. We note that the embeddings matrix E is

bioRxiv preprint doi: https://doi.org/10.64898/2025.12.04.692436; this version posted December 5, 2025. The copyright holder for this preprint (which was not certified by peer review) is the author/funder, who has granted bioRxiv a license to display the preprint in perpetuity. It is made available under a CC-BY 4.0 International license.

already zero-centered dimension-wise (column-wise). That is,

$$\mathbf{1}^T E = \mathbf{0}.$$

This is because:

$$\begin{aligned}\left(\mathbf{1}^T E\right)\left(\mathbf{1}^T E\right)^T &= \mathbf{1}^T E E^T \mathbf{1} \\ &= \mathbf{1}^T V \Lambda V^T \mathbf{1} \\ &= \mathbf{1}^T H C(\mathcal{T}) H \mathbf{1} \\ &= 0.\end{aligned}$$

The covariance matrix for PCA is:

$$\begin{aligned}E^T E &= \Lambda^{\frac{1}{2}} V^T V \Lambda^{\frac{1}{2}} \\ &= \Lambda.\end{aligned}$$

Therefore, the eigenvalues of $E^T E$ are just the diagonal elements of Λ (the eigenvalues of the double-centered variance-covariance matrix), and the eigenvectors are just the standard basis vectors $\{e_i \in \mathbb{R}^n\}$.

Now we show that using the projections of the embeddings of the tips onto the PC-axes is equivalent to using the eigenvectors of the double-centered variance-covariance matrix. Following what we have shown above, the projection of the embeddings of the tips onto the d-th PC-axis is Ee_d. Therefore, we only need to show that Ee_d is the d-th leading eigenvector of doubled-centered variance-covariance matrix:

$$\begin{aligned}V \Lambda V^T E e_d &= E E^T E e_d \\ &= \lambda_d E e_d.\end{aligned}$$

5 Notes on using non-centered variance-covariance matrix

Above we have shown the correctness of using eigenvectors of a double-centered variance-covariance matrix $\breve{C}$ to correct for confounding in phylogenetic regression. However, in practice, we can show that even though using a non-centered variance-covariance matrix C would generate different eigenvectors, in general it can lead to similar results.

As we have discussed in both Section 2 and 4, the double-centered $\breve{C}$ matrix for a tree with n tips is equivalent to the variance-covariance matrix of n points in $\mathbb{R}^n$ space (relative to the mean embedding). The non-centered C then would be the variance-covariance matrix of n points in $\mathbb{R}^n$ space relative to the original point $\mathbf{0}$. Therefore, getting the eigenvectors of the non-centered C would have the same effect as not-centering the data when performing PCA. In PCA, if one does not perform mean-centering on the data,

bioRxiv preprint doi: https://doi.org/10.64898/2025.12.04.692436; this version posted December 5, 2025. The copyright holder for this preprint (which was not certified by peer review) is the author/funder, who has granted bioRxiv a license to display the preprint in perpetuity. It is made available under a CC-BY 4.0 International license.

a large part of the variance from the origin would come from the mean vector, so the first few eigenvectors would be strongly biased by the mean of the data, and the eigenvalues would be overestimated.

However, eigenvectors corresponding to smaller eigenvalues are in general orthogonal to the mean vector, and are thus less affected by the mean-centering transformation. For these smaller eigenvalues, we observe that in general the i-th eigenvalue and eigenvector pair of a double-centered $\breve{C}$ is similar to the $(i+1)$-th eigenvalue and eigenvector pair of a non-centered C. The last eigenvalue and eigenvector pair (0 and $\mathbf{1}$) of the doubled-centered $\breve{C}$ does not have a corresponding pair in the non-centered C, because an eigenvalue of 0 means that the last eigenvector does not explain any variance among the data.

Even though we cannot verify the observation above mathematically, we can think about this in terms of matrix perturbation theory. The difference between the double-centered $\breve{C}$ and the non-centered C is:

$$\begin{aligned}\Delta C(\mathcal{T}) &= HC(\mathcal{T})H - C(\mathcal{T}) \\ &= HC(\mathcal{T})H - C(\mathcal{T})H + C(\mathcal{T})H - C(\mathcal{T}) \\ &= (H - I)\, C(\mathcal{T})H + C(\mathcal{T})\,(H - I) \\ &= -\frac{1}{n}\mathbf{1}\mathbf{1}^T C(\mathcal{T})H - \frac{1}{n}C(\mathcal{T})\mathbf{1}\mathbf{1}^T.\end{aligned}$$

Note that $\mathbf{1}\mathbf{1}^T$ is a rank-1 matrix, so the two components involving $\mathbf{1}\mathbf{1}^T$ in the equation above also give rise to matrices of rank 1, and thus $\Delta C(\mathcal{T})$ is a matrix of rank of at most 2, and at least $n-2$ of its eigenvalues are 0. In fact, we can even show that the matrix has either rank 2 with one positive and one negative eigenvalue or rank 1 with one negative eigenvalue. This is equivalent to showing that matrix $M = \mathbf{1}\mathbf{1}^T C(\mathcal{T})H + C(\mathcal{T})\mathbf{1}\mathbf{1}^T$ has either rank 2 with one positive and one negative eigenvalue or rank 1 with one positive eigenvalue.

First, we can show that M always has one positive eigenvalue using Rayleigh quotient. Let $\boldsymbol{x} = \mathbf{1}$. Then we have:

$$\begin{aligned}\boldsymbol{x}^T M \boldsymbol{x} &= \mathbf{1}^T \left(\mathbf{1}\mathbf{1}^T C(\mathcal{T})H + C(\mathcal{T})\mathbf{1}\mathbf{1}^T\right)\mathbf{1} \\ &= \mathbf{1}^T\mathbf{1}\mathbf{1}^T C(\mathcal{T})H\mathbf{1} + \mathbf{1}^T C(\mathcal{T})\mathbf{1}\mathbf{1}^T\mathbf{1} \\ &= n\mathbf{1}^T C(\mathcal{T})\mathbf{1}.\end{aligned}$$

The Rayleigh quotient would be:

$$\begin{aligned}R\,(M, \boldsymbol{x}) &= \frac{\boldsymbol{x}^T M \boldsymbol{x}}{\boldsymbol{x}^T \boldsymbol{x}} \\ &= \frac{n\mathbf{1}^T C(\mathcal{T})\mathbf{1}}{\mathbf{1}^T\mathbf{1}} \\ &= \mathbf{1}^T C(\mathcal{T})\mathbf{1} \\ &> 0,\end{aligned}$$

bioRxiv preprint doi: https://doi.org/10.64898/2025.12.04.692436; this version posted December 5, 2025. The copyright holder for this preprint (which was not certified by peer review) is the author/funder, who has granted bioRxiv a license to display the preprint in perpetuity. It is made available under a CC-BY 4.0 International license.

where we have the last line because $C(\mathcal{T})$ has non-negative entries and is not all zeroes. We know that:

$$R(M, \boldsymbol{x}) \in [\lambda_{\text{min}}, \lambda_{\text{max}}].$$

Therefore, we know that matrix M has at least one positive eigenvalue. Now we show that matrix M has at least one negative eigenvalue. We note that:

$$\begin{aligned}
tr(M) &= tr\left(\mathbf{1}\mathbf{1}^T C(\mathcal{T})H + C(\mathcal{T})\mathbf{1}\mathbf{1}^T\right) \\
&= tr\left(\mathbf{1}\mathbf{1}^T C(\mathcal{T})H\right) + tr\left(C(\mathcal{T})\mathbf{1}\mathbf{1}^T\right) \\
&= tr\left(H\mathbf{1}\mathbf{1}^T C(\mathcal{T})\right) + tr\left(C(\mathcal{T})\mathbf{1}\mathbf{1}^T\right) \\
&= tr\left(C(\mathcal{T})\mathbf{1}\mathbf{1}^T\right) \\
&= \mathbf{1}^T C(\mathcal{T})\mathbf{1}.
\end{aligned}$$

We also have:

$$tr(M) = \sum_d \lambda_d(M).$$

Then, we have:

$$\sum_d \lambda_d(M) = \mathbf{1}^T C(\mathcal{T})\mathbf{1} = R(M, \boldsymbol{x}) \leq \lambda_{\text{max}}(M).$$

The equality above holds if and only if M has rank 1. Otherwise, M must have at least one negative eigenvalue such that the sum of all the eigenvalues add up to a value less than its largest eigenvalue. And since M has at most a rank of 2, in this case, M would have exactly one positive and one non-positive eigenvalue. Therefore, if we order the eigenvalues $\lambda_1, \lambda_2, \ldots, \lambda_n$ of $\Delta C(\mathcal{T})$ in decreasing order, we obtain:

$$\lambda_1 \geq 0, \lambda_2 = \lambda_3 = \cdots = \lambda_{n-1} = 0, \lambda_n < 0.$$

By Weyl's Inequalities [8], we have:

$$\begin{aligned}
&\lambda_{i+j-1}(A+B) \leq \lambda_i(A) + \lambda_j(B), && i+j \leq n+1, \\
&\lambda_i(A) + \lambda_j(B) \leq \lambda_{i+j-n}(A+B), && i+j \geq n+1,
\end{aligned}$$

where $\lambda_i(A)$ is the i-th largest eigenvalue of matrix A. Let A be the non-centered $C(\mathcal{T})$ and B be $\Delta C(\mathcal{T})$. Since $\lambda_j(\Delta C(\mathcal{T}))$ is 0 for all $j = 2, 3, \ldots, n-1$, we choose $j = 2$ for the first inequality and $j = n-1$

bioRxiv preprint doi: https://doi.org/10.64898/2025.12.04.692436; this version posted December 5, 2025. The copyright holder for this preprint (which was not certified by peer review) is the author/funder, who has granted bioRxiv a license to display the preprint in perpetuity. It is made available under a CC-BY 4.0 International license.

for the second. Then we can show that:

$$\begin{aligned}
\lambda_{i+1}\left(\breve{C}(\mathcal{T})\right) &\leq \lambda_i\left(C(\mathcal{T})\right), && 1 \leq i \leq n-1, \\
\lambda_{i+1}\left(C(\mathcal{T})\right) &\leq \lambda_i\left(\breve{C}(\mathcal{T})\right), && 1 \leq i \leq n-1.
\end{aligned}$$

This sandwich pattern of the eigenvalues of C and $\breve{C}$ indicates that for later dimensions, where the eigenvalues are typically small, not double-centering the matrix C would not impact the eigenvalues by much.

The last piece of the puzzle comes from Equation (18) from our previous paper [7], where we have:

$$\hat{\beta}_x^{(GLS,EIG)} = \frac{\sum_{d=J+1}^{n} \lambda_d^{-1}\left(v_d^T x\right)\left(v_d^T y\right)}{\sum_{d=J+1}^{n} \lambda_d^{-1}\left(v_d^T x\right)\left(v_d^T x\right)}.$$

Therefore, even when the eigenvectors of a non-centered variance-covariance matrix are used in phylogenetic regressions, when getting rid of the first J eigenvectors, one also gets rid of the eigenvectors that are the most biased by mean of the matrix. Since the remaining eigenvalue and eigenvector pairs of the non-centered variance-covariance matrix are similar to the one of the double-centered variance-covariance matrix, the estimate of β in the analyses would not deviate much from the estimate using a double-centered variance-covariance matrix.

6 Overall contributions of each branch

Once the contributions of the branches to each of the eigenvectors are obtained, we can also compute the overall contributions of each branch. Recall that $w_d(e)$ is the contribution of some branch e of a phylogenetic tree to the d-th eigenvector of some version of variance-covariance matrix of the tree. Then the overall contribution of the branch e across all the dimensions would be:

$$w(e) = \sqrt{\sum_{d=1}^{n} \lambda_d w_d^2(e)} \tag{13}$$

where λ_d is the d-th eigenvalue of the variance-covariance matrix. Note that the formula looks very much like a Euclidean norm, except that each dimension is also weighted by the corresponding eigenvalue. We choose the Euclidean norm for two reasons: (1) the eigenvectors represent dimensions orthogonal to each other, which makes the Euclidean norm appropriate, (2) $w_d(e)$ can be negative, so directly adding up the contributions across the dimensions could potentially cancel out some of the terms and underestimate the contributions of some branches. We also weight each dimension by λ_d because the first few dimensions explain the most variance and are thus more important than the later dimensions. The overall contributions of each branch can be used as a metric for the importance of each branch in the phylogenetic tree.

bioRxiv preprint doi: https://doi.org/10.64898/2025.12.04.692436; this version posted December 5, 2025. The copyright holder for this preprint (which was not certified by peer review) is the author/funder, who has granted bioRxiv a license to display the preprint in perpetuity. It is made available under a CC-BY 4.0 International license.

7 Connection between PGLS and PGLMM

We use PGLS and PGLMM interchangeably throughout the discussion in our paper, because they are equivalent in our simulation scenarios. Here we provide the proof. Suppose that we are including the first J eigenvectors as covariates, then in PGLS, we have:

$$y = \beta x + \sum_{d=1}^{J} \beta_d v_d + \varepsilon_{PGLS}, \qquad \varepsilon_{PGLS} \sim \mathcal{N}\left(0, \sigma_G^2 \Sigma + \sigma_E^2 I\right).$$

In PGLMM, we have:

$$y = \beta x + \sum_{d=1}^{J} \beta_d v_d + Zu + \varepsilon_{PGLMM}, \qquad Z = I, u \sim \mathcal{N}\left(0, \sigma_G^2 \Sigma\right), \varepsilon_{PGLMM} \sim \mathcal{N}\left(0, \sigma_E^2 I\right).$$

The reason why the design matrix Z above is equivalent to the identity matrix is that we have only one individual for each species. Then, for both equations above, we can show that y follows the same multivariate normal distribution:

$$y \sim \mathcal{N}(\beta x + \sum_{d=1}^{J} \beta_d v_d, \sigma_G^2 \Sigma + \sigma_E^2 I).$$

The only difference between the two methods is that PGLS is including the variance-covariance matrix Σ in the variance structure of the residuals, whereas PGLMM is including Σ as random effects.

8 Performance of different methods in Felsenstein's Worst Case

In our simulations for Felsenstein's Worst Case (FWC), we observe that when we include v_1 of C as fixed effect, the results for OLS and PGLS are exactly the same. Here we show mathematically why this would be the case. Suppose that we have n species in each clade in FWC, and we assume a Brownian motion rate of 1, then the phylogenetic variance-covariance matrix for the $2n$ species would be:

$$C = l_1 \begin{bmatrix} \mathbf{1}\mathbf{1}^T & \mathbf{0} \\ \mathbf{0} & \mathbf{1}\mathbf{1}^T \end{bmatrix} + l_2 I_{2n},$$

where $\mathbf{1}$ is a one-vector of length n, $\mathbf{0}$ is an $n \times n$ zero-matrix, I_{2n} is the $2n \times 2n$ identity matrix, and l_1 and l_2 are the lengths of the branches outside and within the cade, respectively.

Note that we can rewrite C as:

$$C = \begin{bmatrix} l_1 \mathbf{1}\mathbf{1}^T + l_2 I_n & \mathbf{0} \\ \mathbf{0} & l_1 \mathbf{1}\mathbf{1}^T + l_2 I_n \end{bmatrix} = \begin{bmatrix} C_0 & \mathbf{0} \\ \mathbf{0} & C_0 \end{bmatrix}.$$

bioRxiv preprint doi: https://doi.org/10.64898/2025.12.04.692436; this version posted December 5, 2025. The copyright holder for this preprint (which was not certified by peer review) is the author/funder, who has granted bioRxiv a license to display the preprint in perpetuity. It is made available under a CC-BY 4.0 International license.

Now we show that if λ and v is an eigenvalue-eigenvector pair for C_0, then λ and $\begin{bmatrix} v^T & 0 & 0 & \dots & 0 \end{bmatrix}^T$ and λ and $\begin{bmatrix} 0 & 0 & \dots & 0 & v^T \end{bmatrix}^T$ are two pairs of eigenvalues and eigenvectors for C. This is because:

$$C \begin{bmatrix} v \\ \vec{\mathbf{0}} \end{bmatrix} = \begin{bmatrix} C_0 & \mathbf{0} \\ \mathbf{0} & C_0 \end{bmatrix} \begin{bmatrix} v \\ \vec{\mathbf{0}} \end{bmatrix} = \lambda \begin{bmatrix} v \\ \vec{\mathbf{0}} \end{bmatrix},$$

$$C \begin{bmatrix} \vec{\mathbf{0}} \\ v \end{bmatrix} = \begin{bmatrix} C_0 & \mathbf{0} \\ \mathbf{0} & C_0 \end{bmatrix} \begin{bmatrix} \vec{\mathbf{0}} \\ v \end{bmatrix} = \lambda \begin{bmatrix} \vec{\mathbf{0}} \\ v \end{bmatrix}.$$

Thus, to get the eigenvector decomposition for C, we only need to do the decomposition on C_0. We can easily show that $l_1 n + l_2$ and $\mathbf{1}$ is a pair of eigenvalue and eigenvector for C_0, since:

$$\left(l_1 \mathbf{1}\mathbf{1}^T + l_2 I_n\right) \mathbf{1} = (l_1 n + l_2)\,\mathbf{1}.$$

Similarly, we can show that l_2 and any vector v such that $v^T\mathbf{1} = 0$ is a pair of eigenvalue and eigenvector for C_0, since:

$$\left(l_1 \mathbf{1}\mathbf{1}^T + l_2 I_n\right) v = l_2 v.$$

There would be a total of $n-1$ such eigenvectors v in $\mathbb{R}^n$ space. Thus, the eigenvalues of C_0 are $l_1 n + l_2$, l_2, l_2, ..., l_2, and the eigenvalues of C are just the duplicates of these eigenvalues. Now, for OLS with v_1 as covariate, our design matrix would be $X = \begin{bmatrix} \mathbf{1} & x & v_1 \end{bmatrix}$, which is slightly different from the formula used to derive equation (16) in our previous paper [7]. However, we observe that $y = \beta_0 + \beta_x x + \beta_1 v_1 + \varepsilon_{OLS}$ is equivalent to $y = \beta_x x + \beta_1 v_1 + \beta_2 v_2 + \varepsilon_{OLS}$, since:

$$\begin{aligned} v_1 + v_2 &= \frac{1}{\sqrt{n}} \begin{bmatrix} 1 & 1 & \dots & 1 & 0 & 0 & \dots & 0 \end{bmatrix}^T + \frac{1}{\sqrt{n}} \begin{bmatrix} 0 & 0 & \dots & 0 & 1 & 1 & \dots & 1 \end{bmatrix}^T \\ &= \frac{1}{\sqrt{n}} \mathbf{1}. \end{aligned}$$

That is, including the first eigenvector and the intercept as fixed effects in FWC is the same as including the first two eigenvectors without the intercept. And thus we have:

$$\hat{\beta}_x^{(OLS,EIG)} = \frac{\sum_{d=3}^{2n} \left(v_d^T x\right)\left(v_d^T y\right)}{\sum_{d=3}^{2n} \left(v_d^T x\right)\left(v_d^T x\right)}.$$

bioRxiv preprint doi: https://doi.org/10.64898/2025.12.04.692436; this version posted December 5, 2025. The copyright holder for this preprint (which was not certified by peer review) is the author/funder, who has granted bioRxiv a license to display the preprint in perpetuity. It is made available under a CC-BY 4.0 International license.

Similarly, we can plug in equation (18) in our previous paper [7] using the same trick for PGLS:

$$\begin{aligned}\hat{\beta}_x^{(PGLS,EIG)} &= \frac{\sum_{d=3}^{2n} \lambda_d^{-1} \left(v_d^T x\right) \left(v_d^T y\right)}{\sum_{d=3}^{2n} \lambda_d^{-1} \left(v_d^T x\right) \left(v_d^T x\right)} \\ &= \frac{\sum_{d=3}^{2n} \left(v_d^T x\right) \left(v_d^T y\right)}{\sum_{d=3}^{2n} \left(v_d^T x\right) \left(v_d^T x\right)} \\ &= \hat{\beta}_x^{(OLS,EIG)},\end{aligned}$$

where the second row is obtained by plugging in $v_d = l_2$ for all $d \geq 3$. Therefore, we have shown using mathematical analyses why we would obtain exactly the same results for OLS and PGLS when including the first eigenvector and the intercept as covariates.

9 Supplementary Methods

In the sections above, we have addressed multiple matrices that can be used to capture the relationships between species of a phylogenetic tree, so we first compared how branches contribute to the eigenvectors of these different matrices, including expected genetic relatedness matrix (eGRM, or Σ), phylogenetic variance-covariance matrix (VCV, or C), and its double-centered version (dcVCV, or $\breve{C}$). We specifically investigated three different types of trees. For Felsenstein's Worst Case (FWC), we checked how the branches contribute to v_1 of these different matrices (Fig. S1). We then looked at the contributions of branches to the first three eigenvectors of these different matrices for a 10-species Yule tree (Fig. S2) and a 10-species caterpillar tree (Fig. S3).

While we have shown in the sections above that it is theoretically rigorous to use the double-centered version $\breve{C}$ to get the eigenvectors, we also want to know how using a non-centered C in practice might change the results of phylogenetic regressions. To compare the performance of using C and $\breve{C}$ to get the eigenvectors, we simulated Yule trees, caterpillar trees, fully balanced trees, and coalescent trees of 128 species. For each tree type, we generated 500 different ultrametric trees with varying branch lengths (Fig. S4). Then we simulated x and y as arising independently from a Brownian motion along each of the trees. We compared the performance of using different numbers of eigenvectors of C and $\breve{C}$ in OLS and PGLS for phylogenetic regression with `R` function `lm()` and `phylolm()` from the package `phylolm` [4, 5].

We also checked the performance of using the eigenvectors of a non-centered C in phylogenetic regressions for non-ultrametric trees, where we followed similar procedure as in the ultrametric case, but we used pure caterpillar trees, fully balanced trees, and moderately imbalanced trees of 128 species instead (Fig. S5). The moderately imbalanced trees were simulated by first generating coalescent trees and then reassigning branch lengths as independent exponential random variables Exp(1).

Similar to Fig. 2, we tested the effect of tree size on the performance of OLS and PGLS with eigenvectors in non-ultrametric trees. We simulated caterpillar trees, fully balanced trees, and moderately imbalanced

bioRxiv preprint doi: https://doi.org/10.64898/2025.12.04.692436; this version posted December 5, 2025. The copyright holder for this preprint (which was not certified by peer review) is the author/funder, who has granted bioRxiv a license to display the preprint in perpetuity. It is made available under a CC-BY 4.0 International license.

trees of 8, 16, 32, 64, 128, 256, 512, and 1024 species (Fig. S6). For each combination of tree type and tree size, we generated 500 different non-ultrametric trees with varying branch lengths. The rest of the procedure is exactly the same as in previous simulations.

To understand the number of eigenvectors we need to include as covariates in the model to control for confounding when there are non-Brownian shifts on a phylogenetic tree, we generated the same 16-species Yule tree as in Fig. 4, and we visualized the contributions of branches to the first eight eigenvectors of the phylogenetic variance-covariance matrix C (Fig. S7). We first looked at cases when there is only one non-Brownian shift on the tree. The simulation procedure is exactly the same as is mentioned in the Methods section for the main text. For each combination of shift position, shift type, and shift size, we ran a total of 100 replicates (Figs. S8 and S9).

We further investigated how the performance of PGLS with no eigenvectors can be impacted by where the shift falls on the tree. We generated a total of 500 Yule trees of 16 species. For each tree, we first calculated the overall contributions of the branches across the dimensions using equation (13) from Section 6, with which we ranked the branches in decreasing order. Then we simulated x and y following the same procedure, and for each combination of shift type and size, we checked how the false positive rate of PGLS changes with the rank of the branch where the shift occurs (Fig. S10).

Next we turned to investigate the cases when there are two non-Brownian shifts on the tree. We used the same 16-species Yule tree as in the cases for one non-Brownian shift in Fig. S7. Similar to previous simulations, we first simulated x and y as arising independently from a Brownian motion along each of the trees. Then, out of the nine branches that contribute mostly to the first eight eigenvectors of the C matrix (branches labeled 1, 2, 11, 15, 17, 23, 24, 25, and 26 in Fig. S7), we selected two branches when the shifts occur. Then we evaluated the performance of using different numbers of eigenvectors of C in PGLS. For each combination of shift position pairs, shift type, and shift size, we ran a total of 100 replicates (Figs. S11 and S12).

We also analyzed the cases when there are multiple non-Brownian shifts on the same 16-species Yule tree as in Fig. S7. We specifically looked at the cases when there are shifts on 4 branches (labeled 1, 2, 23, and 25 in Fig. 7), when there are shifts on 8 branches (labeled as 1, 2, 11, 15, 17, 23, 25, and 26), and when there shifts on 16 branches (labeled as 1, 2, 9, 10, 11, 15, 17, 18, 23, 24, 25, and 26). We ran the analyses using the same simulation procedure as in the cases for two shifts, and we ran 100 replicates for each combination of shift positions, shift type, and shift size (Fig. S13).

While we focused mostly on how the combined method helps mitigate confounding to reduce false positives in phylogenetic regressions in all the previous simulations, we are also interested in the performance of the combined method in terms of power. We first compared the performance of using the eigenvectors of C and $\breve{C}$ in OLS and PGLS. We simulated Yule trees, caterpillar trees, fully balanced trees, and coalescent trees of 128 species. For each tree type, we generated 500 different ultrametric trees with varying branch lengths. Then we simulated x as arising from a Brownian motion along each of the trees, and y as the combination of a dependent part βx and an independent part y_0, where β, the true regression coefficient, was set to be

bioRxiv preprint doi: https://doi.org/10.64898/2025.12.04.692436; this version posted December 5, 2025. The copyright holder for this preprint (which was not certified by peer review) is the author/funder, who has granted bioRxiv a license to display the preprint in perpetuity. It is made available under a CC-BY 4.0 International license.

0.5, and y_0 was simulated as arising from a Brownian motion along the tree independent from x. Then we compared the recall ($1 -$ False Negative Rate) of using different numbers of eigenvectors of C and $\breve{C}$ in OLS and PGLS for phylogenetic regressions (Fig. S14).

Next we explored the effect of tree size and β on the recall of using different numbers of eigenvectors in OLS and PGLS. We simulated Yule trees of 8, 16, 32, 64, 128, 256, 512, and 1024 species (Fig. S15). Then we simulated x as arising from a Brownian motion along each of the trees, and y as the combination of a dependent part βx and an independent part y_0, where β, the true regression coefficient, was set to be some value from 0.0625 to 16, and y_0 was simulated as arising from a Brownian motion along the tree independent from x. Then we compared the recall of using different numbers of eigenvectors of C in OLS and PGLS. For each combination of tree size and β, we ran a total of 100 different replicates.

Finally, we looked at the recall when there is also a non-Brownian shift on the tree, and we used the same 16-species Yule tree as in Fig. S7. We focused on a subset of branches where the shifts may occur (labeled as 1, 2, 23, and 25 in Fig. S7). We simulated x as arising from a Brownian motion along the tree and generated the shift by adding a normal random variable (or a constant) to the species that are descendants of the branch where the shift occurs. Then we simulated y as the combination of a dependent part βx and an independent part y_0, where β, the true regression coefficient, was set to be some value from 0.0625 to 2, and y_0 was simulated as arising from a Brownian motion along the tree plus some shift, both of which are independent from x. Next we compared the recall of using different numbers of eigenvectors of C in OLS and PGLS, and for each combination of shift position, shift type, shift size, and β, we ran a total of 100 different replicates (Figs. S16 and S17).

bioRxiv preprint doi: https://doi.org/10.64898/2025.12.04.692436; this version posted December 5, 2025. The copyright holder for this preprint (which was not certified by peer review) is the author/funder, who has granted bioRxiv a license to display the preprint in perpetuity. It is made available under a CC-BY 4.0 International license.

10 Supplementary Figures

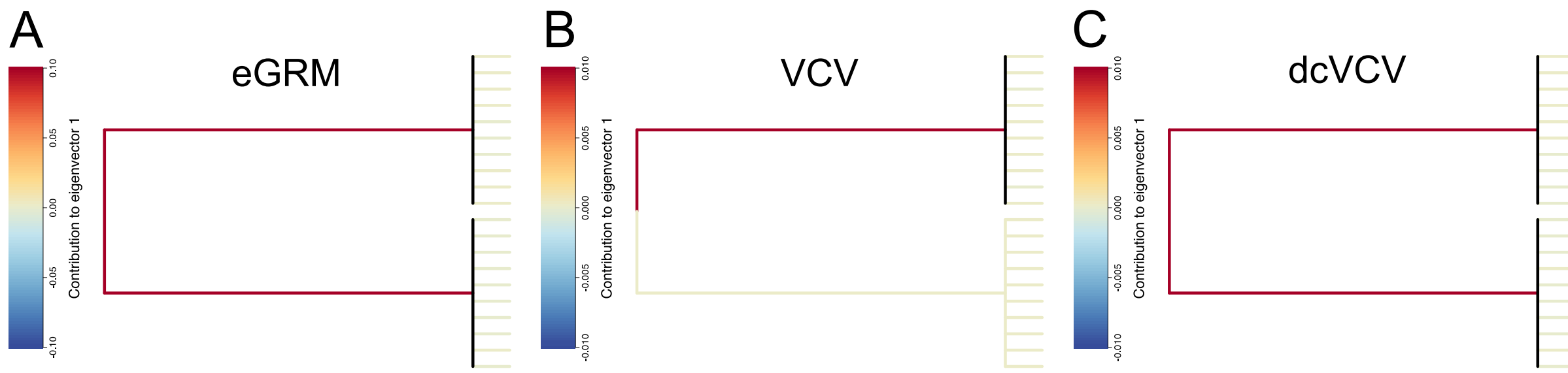

Fig S1: Contributions of branches to the first eigenvector v_1 of different matrices of a phylogenetic tree depicting Felsenstein's Worst Case (FWC). Each clade contains 10 species. (A) Contributions of branches to v_1 of the expected genetic relatedness matrix (Σ) of the phylogenetic tree depicting FWC. (B) Contributions of branches to v_1 of the non-centered phylogenetic variance-covariance matrix (C) of the phylogenetic tree depicting FWC. (C) Contributions of branches to v_1 of the double-centered phylogenetic variance-covariance matrix ($\breve{C}$) of the phylogenetic tree depicting FWC. The vertical branches are black because the tip branches slightly differ in the their contributions to v_1.

bioRxiv preprint doi: https://doi.org/10.64898/2025.12.04.692436; this version posted December 5, 2025. The copyright holder for this preprint (which was not certified by peer review) is the author/funder, who has granted bioRxiv a license to display the preprint in perpetuity. It is made available under a CC-BY 4.0 International license.

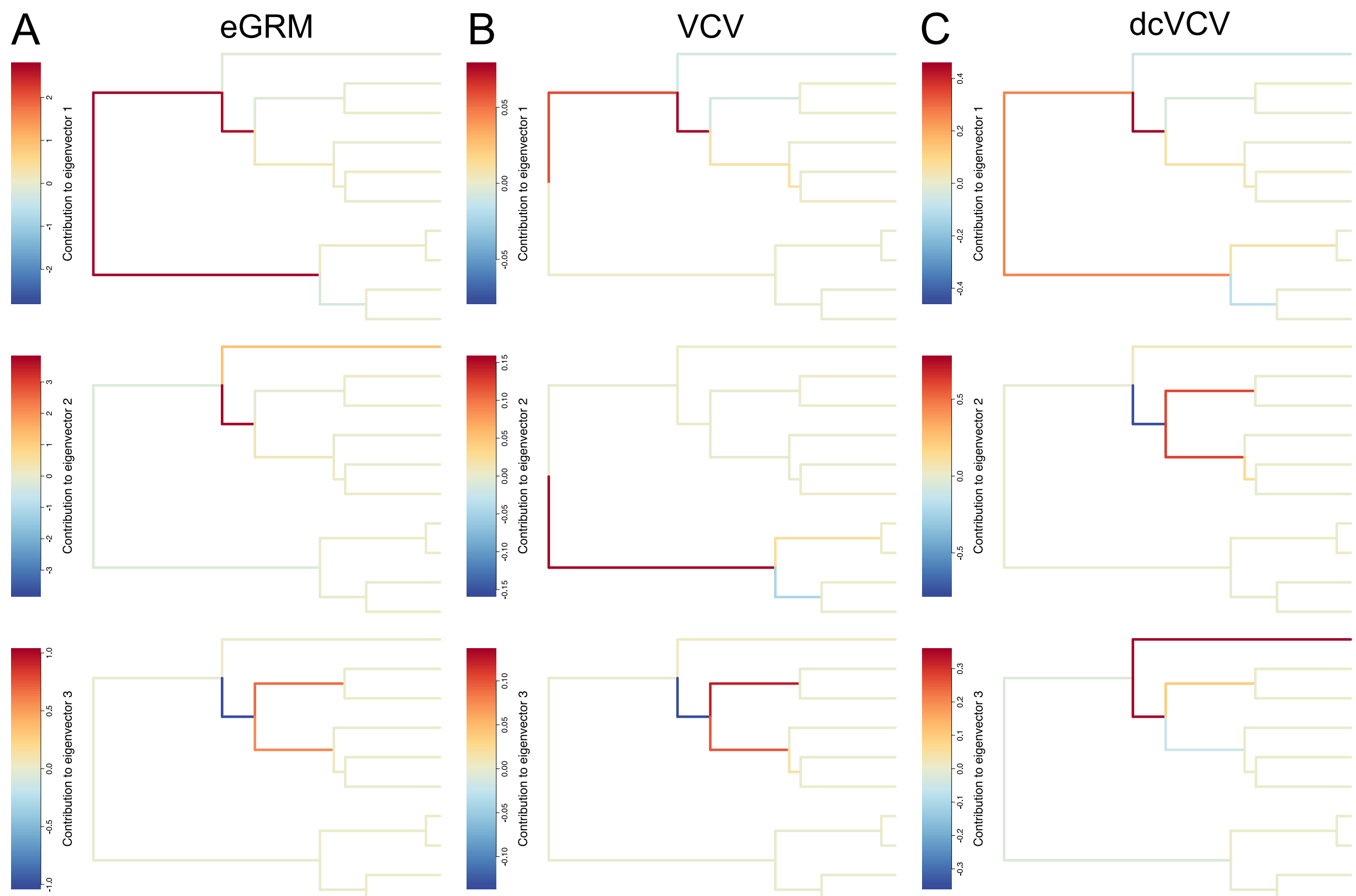

Fig S2: Contributions of branches to the first three eigenvectors of different matrices of a Yule tree containing 10 species. (A) Contributions of branches to the first three eigenvectors of the expected genetic relatedness matrix (Σ) of the Yule tree. (B) Contributions of branches to the first three eigenvectors of the non-centered phylogenetic variance-covariance matrix (C) of the Yule tree. (C) Contributions of branches to the first three eigenvectors of the double-centered phylogenetic variance-covariance matrix ($\breve{C}$) of the Yule tree.

bioRxiv preprint doi: https://doi.org/10.64898/2025.12.04.692436; this version posted December 5, 2025. The copyright holder for this preprint (which was not certified by peer review) is the author/funder, who has granted bioRxiv a license to display the preprint in perpetuity. It is made available under a CC-BY 4.0 International license.

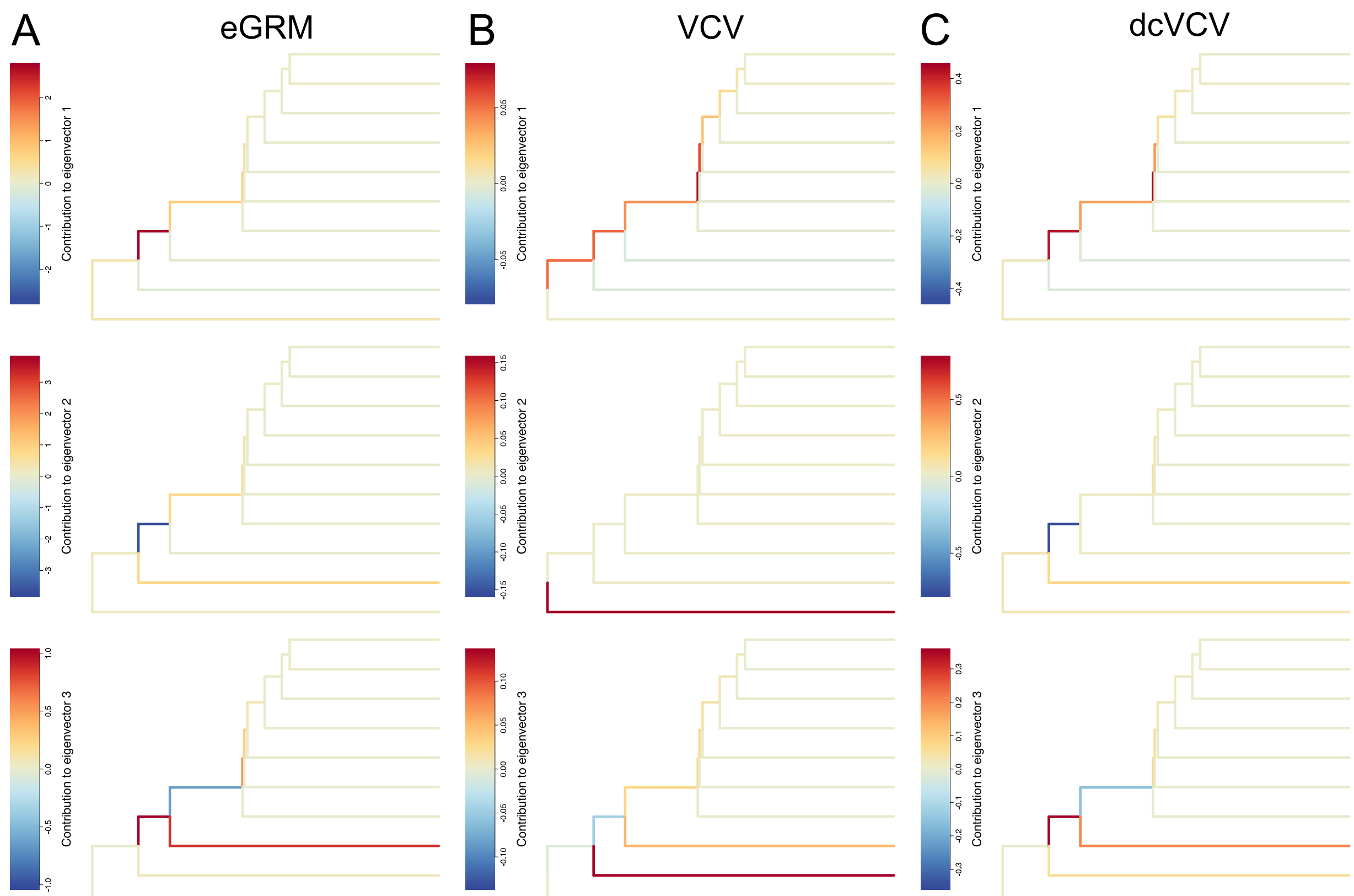

Fig S3: Contributions of branches to the first three eigenvectors of different matrices of a caterpillar tree containing 10 species. (A) Contributions of branches to the first three eigenvectors of the expected genetic relatedness matrix (Σ) of the caterpillar tree. (B) Contributions of branches to the first three eigenvectors of the non-centered phylogenetic variance-covariance matrix (C) of the caterpillar tree. (C) Contributions of branches to the first three eigenvectors of the double-centered phylogenetic variance-covariance matrix ($\breve{C}$) of the caterpillar tree.

bioRxiv preprint doi: https://doi.org/10.64898/2025.12.04.692436; this version posted December 5, 2025. The copyright holder for this preprint (which was not certified by peer review) is the author/funder, who has granted bioRxiv a license to display the preprint in perpetuity. It is made available under a CC-BY 4.0 International license.

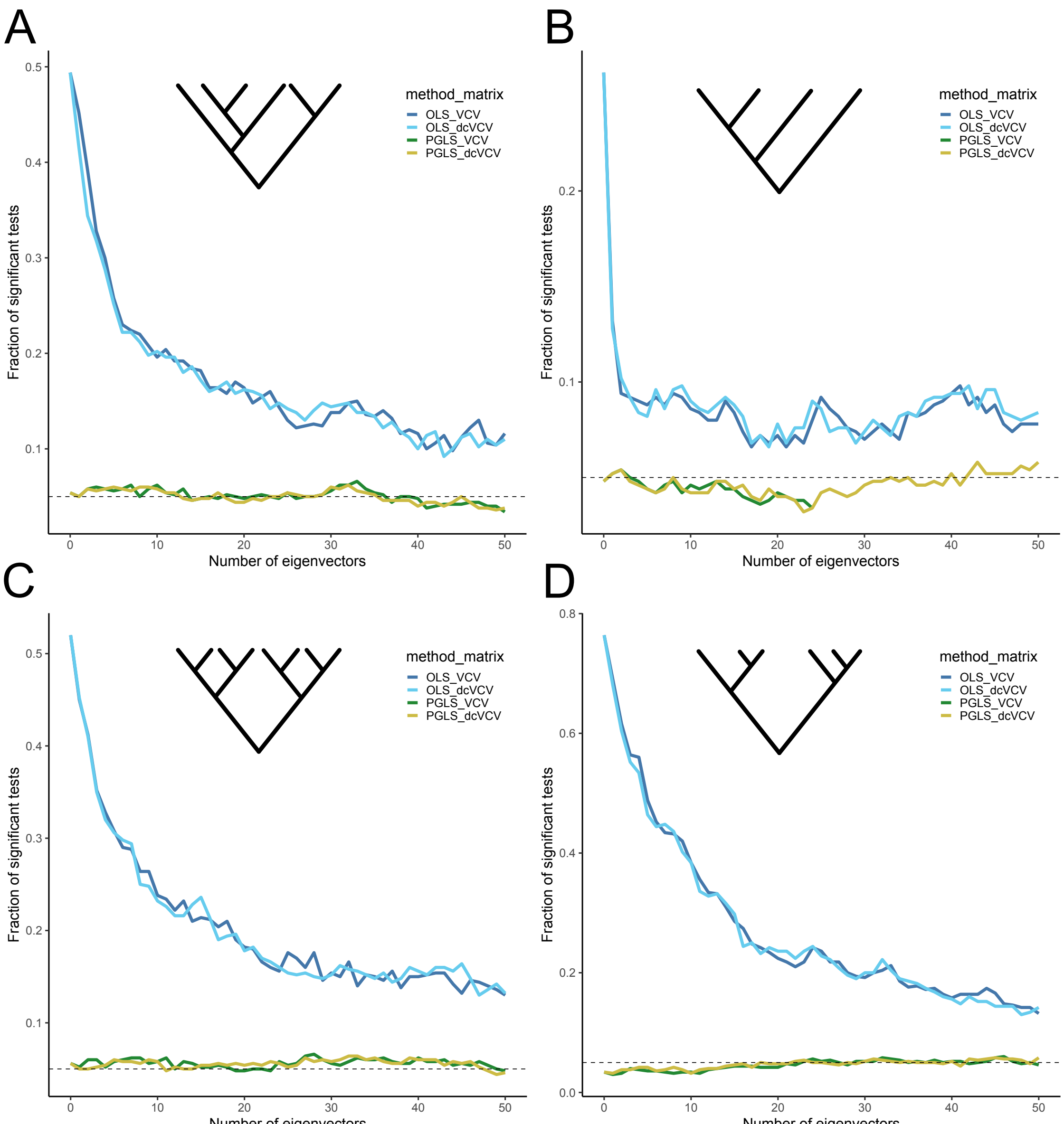

Fig S4: The performance of using eigenvectors of non-centered and double-centered phylogenetic variance-covariance matrices (C and $\breve{C}$) in ordinary least squares (OLS) and phylogenetic generalized least squares (PGLS) to remove phylogenetic confounding for different types of ultrametric trees. (A) Performance of OLS and PGLS with eigenvectors of C and $\breve{C}$ in models related by a Yule tree of 128 species. For simplicity, a Yule tree with only 6 tips is shown. The horizontal axis shows the number of eigenvectors included as covariates, and the vertical axis shows the fraction of tests that would be significant at the 0.05 level. (B) Performance of OLS and PGLS with eigenvectors of C and $\breve{C}$ in models related by a caterpillar tree of 128 species. For simplicity, a caterpillar tree with only 4 tips is shown. The horizontal axis shows the number of eigenvectors included as covariates, and the vertical axis shows the fraction of tests that would be significant at the 0.05 level. (C) Performance of OLS and PGLS with eigenvectors of C and $\breve{C}$ in models related by a fully balanced tree of 128 species. For simplicity, a fully balanced tree with only 8 tips is shown. The horizontal axis shows the number of eigenvectors included as covariates, and the vertical axis shows the fraction of tests that would be significant at the 0.05 level. (D) Performance of OLS and PGLS with eigenvectors of C and $\breve{C}$ in models related by a coalescent tree of 128 species. For simplicity, a coalescent tree with only 6 tips is shown. The horizontal axis shows the number of eigenvectors included as covariates, and the vertical axis shows the fraction of tests that would be significant at the 0.05 level.

bioRxiv preprint doi: https://doi.org/10.64898/2025.12.04.692436; this version posted December 5, 2025. The copyright holder for this preprint (which was not certified by peer review) is the author/funder, who has granted bioRxiv a license to display the preprint in perpetuity. It is made available under a CC-BY 4.0 International license.

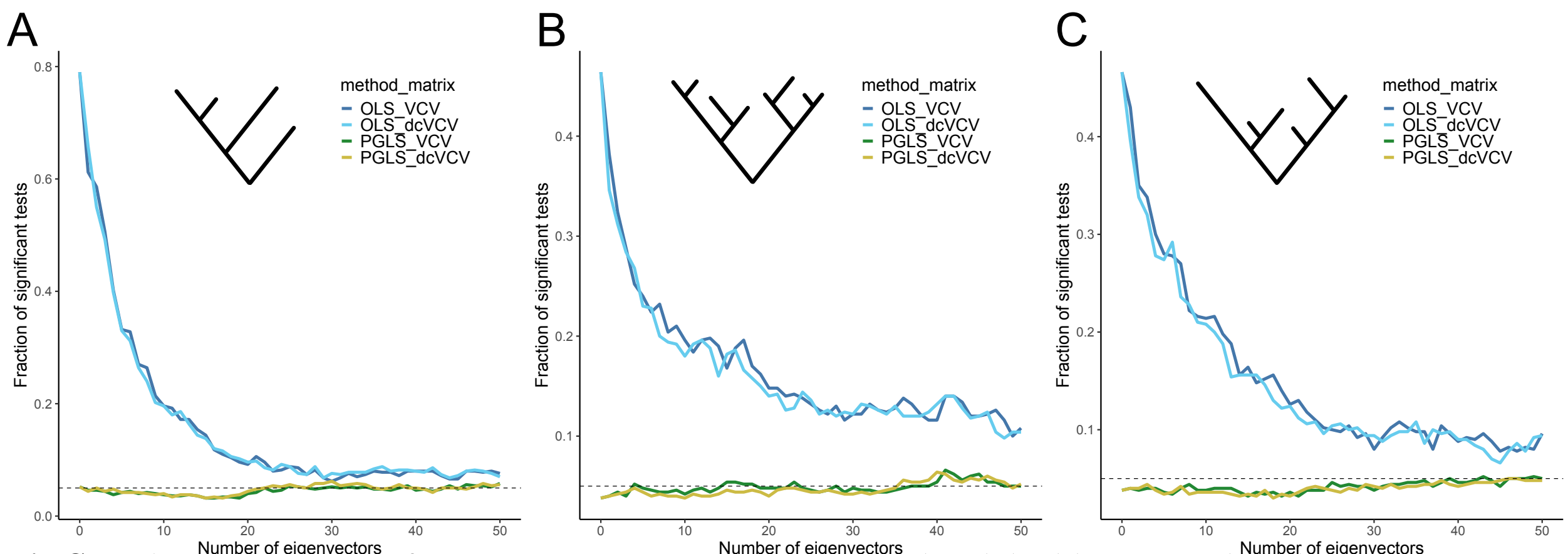

Fig S5: The performance of using eigenvectors of non-centered and double-centered phylogenetic variance-covariance matrices (C and $\breve{C}$) in ordinary least squares (OLS) and phylogenetic generalized least squares (PGLS) to remove phylogenetic confounding for different types of non-ultrametric trees. (A) Performance of OLS and PGLS with eigenvectors of C and $\breve{C}$ in models related by a non-ultrametric caterpillar tree of 128 species. For simplicity, a caterpillar tree with only 4 tips is shown. The horizontal axis shows the number of eigenvectors included as covariates, and the vertical axis shows the fraction of tests that would be significant at the 0.05 level. (B) Performance of OLS and PGLS with eigenvectors of C and $\breve{C}$ in models related by a non-ultrametric fully balanced tree of 128 species. For simplicity, a fully balanced tree with only 4 tips is shown. The horizontal axis shows the number of eigenvectors included as covariates, and the vertical axis shows the fraction of tests that would be significant at the 0.05 level. (C) Performance of OLS and PGLS with eigenvectors of C and $\breve{C}$ in models related by a non-ultrametric moderately imbalanced tree of 128 species. For simplicity, a moderately imbalanced tree with only 6 tips is shown. The horizontal axis shows the number of eigenvectors included as covariates, and the vertical axis shows the fraction of tests that would be significant at the 0.05 level.

bioRxiv preprint doi: https://doi.org/10.64898/2025.12.04.692436; this version posted December 5, 2025. The copyright holder for this preprint (which was not certified by peer review) is the author/funder, who has granted bioRxiv a license to display the preprint in perpetuity. It is made available under a CC-BY 4.0 International license.

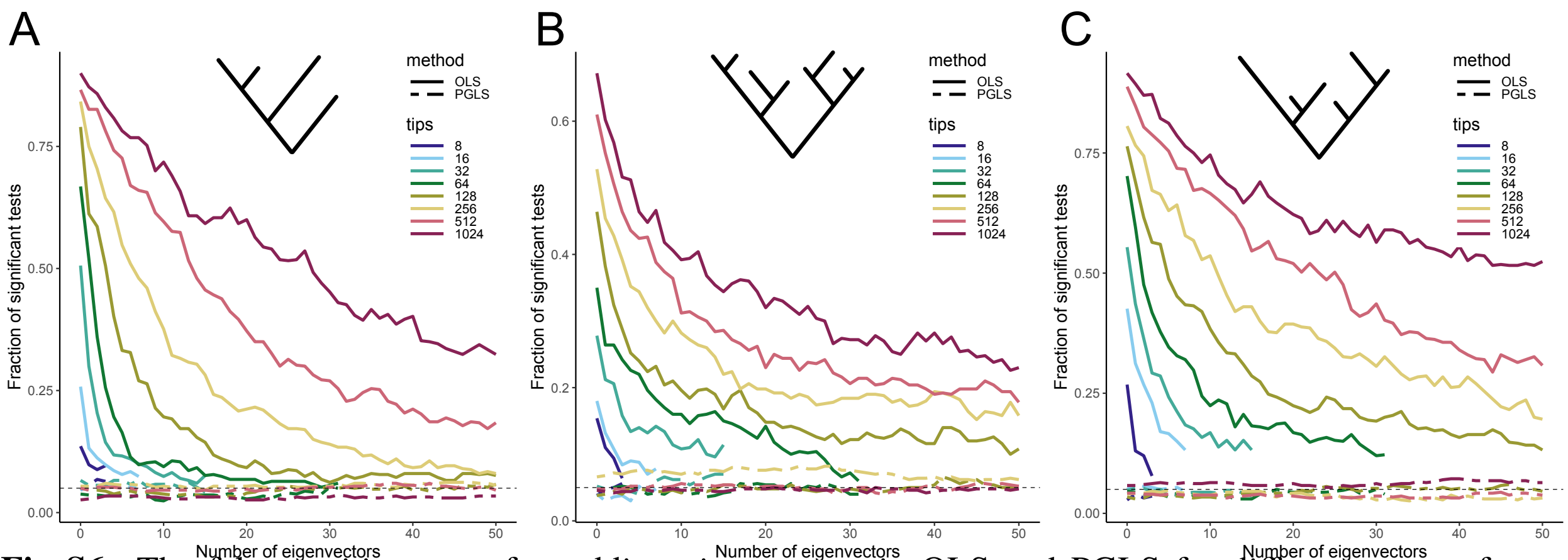

Fig S6: The false positive rate after adding eigenvectors in OLS and PGLS for different types of non-ultrametric trees when the data is generated by a pure Brownian motion with no additional shifts. (A) Performance of OLS and PGLS in models related by a non-ultrametric caterpillar tree of varying numbers of species. For simplicity, a non-ultrametric caterpillar tree with only 4 tips is shown. The horizontal axis shows the number of eigenvectors included as covariates, and the vertical axis shows the fraction of tests that would be significant at the 0.05 level. (B) Performance of OLS and PGLS in models related by a non-ultrametric fully balanced tree of varying numbers of species. For simplicity, a non-ultrametric fully balanced tree with only 8 tips is shown. The horizontal axis shows the number of eigenvectors included as covariates, and the vertical axis shows the fraction of tests that would be significant at the 0.05 level. (C) Performance of OLS and PGLS in models related by a non-ultrametric moderately imbalanced tree of varying numbers of species. For simplicity, a non-ultrametric moderately imbalanced tree with only 6 tips is shown. The horizontal axis shows the number of eigenvectors included as covariates, and the vertical axis shows the fraction of tests that would be significant at the 0.05 level.

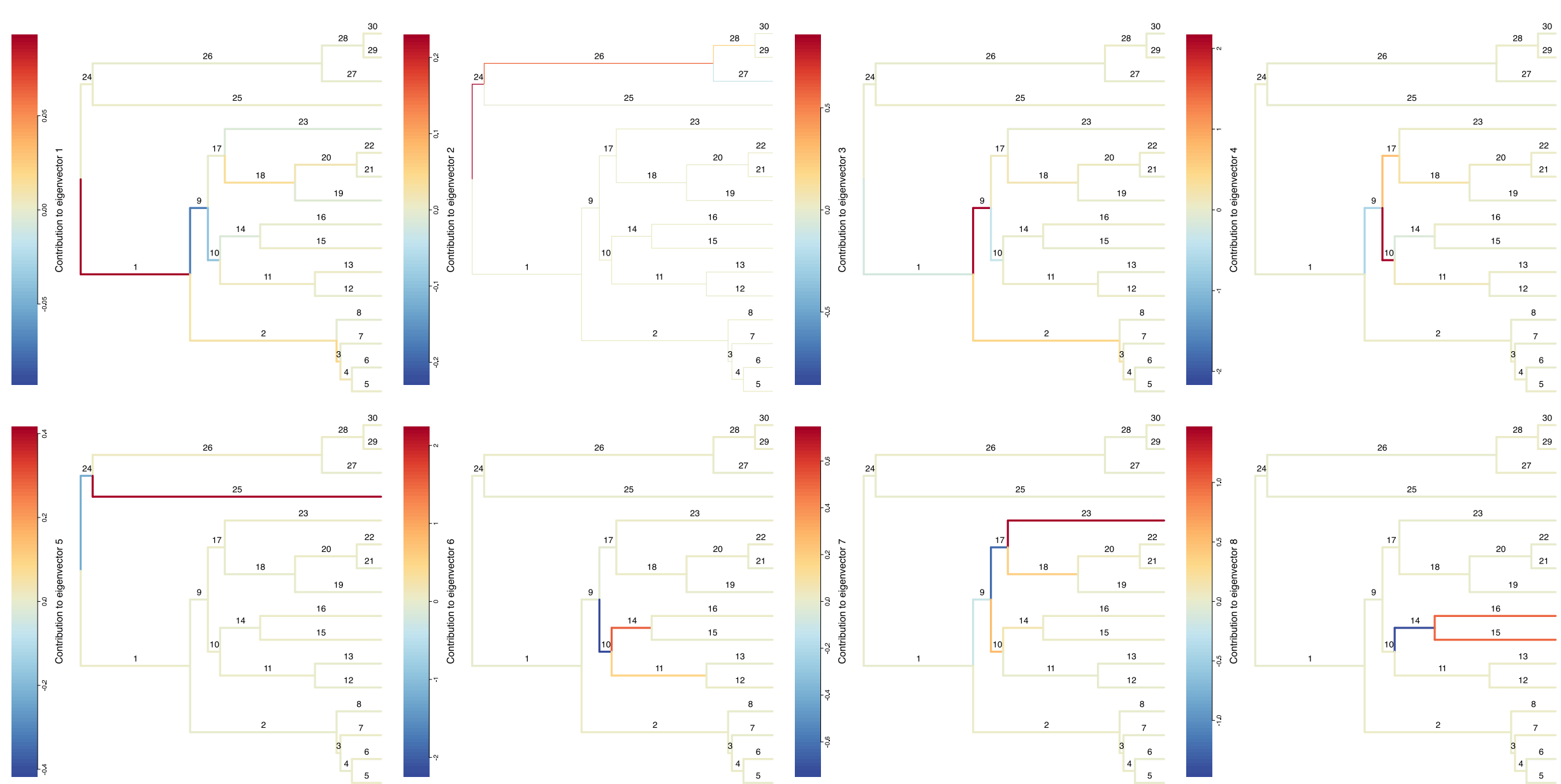

Fig S7: Contributions of branches to the first eight eigenvectors of the phylogenetic variance-covariance matrix C of a Yule tree containing 16 species. The numbers above each branch indicate the labels for the branches.

bioRxiv preprint doi: https://doi.org/10.64898/2025.12.04.692436; this version posted December 5, 2025. The copyright holder for this preprint (which was not certified by peer review) is the author/funder, who has granted bioRxiv a license to display the preprint in perpetuity. It is made available under a CC-BY 4.0 International license.

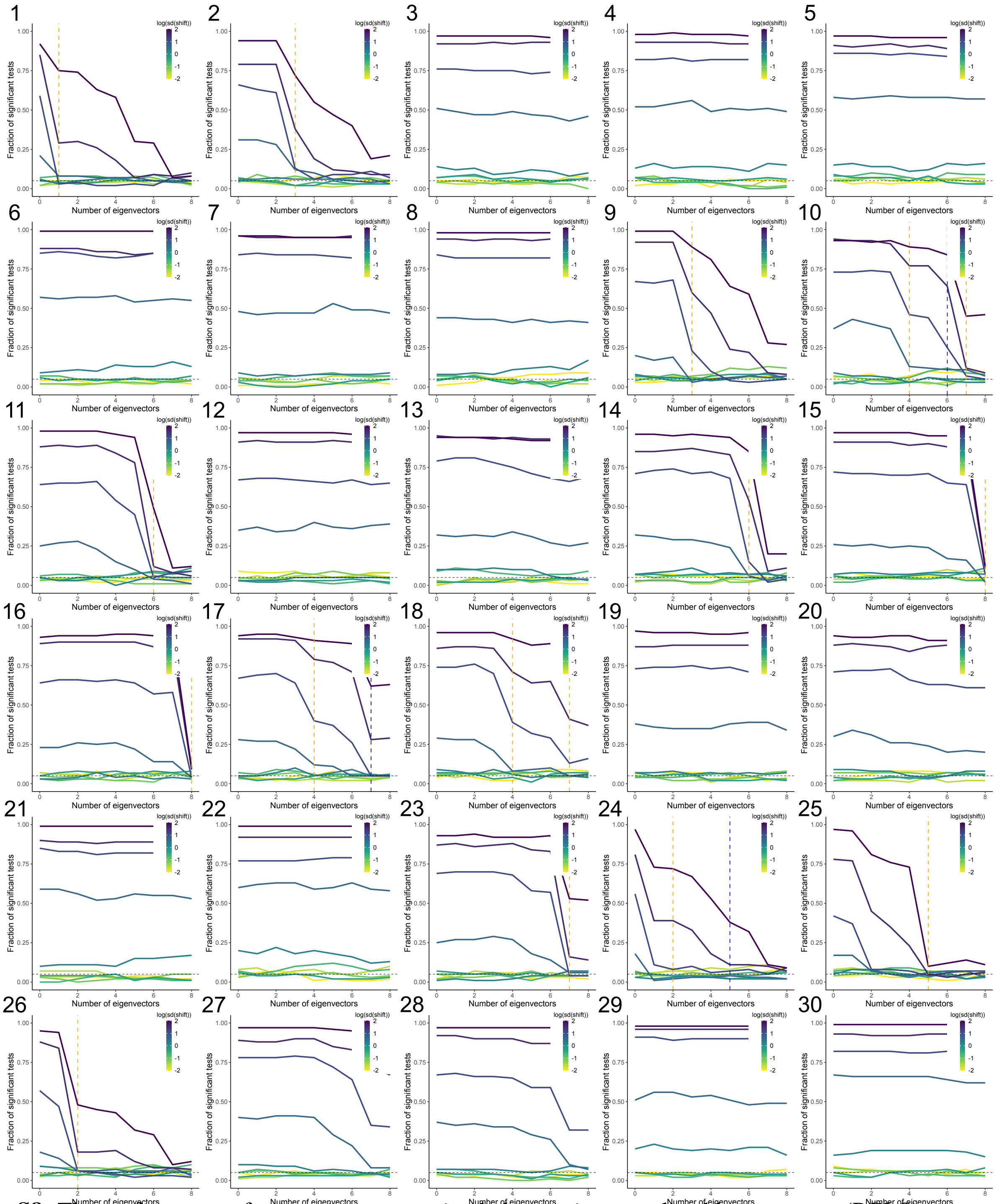

Fig S8: The performance of using eigenvectors in phylogenetic generalized least squares (PGLS) to remove phylogenetic confounding in a model with 16 tips related by a Yule tree with non-Brownian shifts in both predictor and outcome variables. The shift is simulated by adding a normal random variable with varying standard deviation to the species that are descendants of the branch. The label of each panel corresponds to the label of the branch where the shift occurs on the tree in Fig. S7. The horizontal axis shows the number of eigenvectors included as covariates, and the vertical axis shows the fraction of tests that would be significant at the 0.05 level. The vertical orange dashed lines show the dimensions to which the branch concerned makes large positive contributions, and the vertical blue dashed lines show the dimensions to which the branch concerned makes large negative contributions.

bioRxiv preprint doi: https://doi.org/10.64898/2025.12.04.692436; this version posted December 5, 2025. The copyright holder for this preprint (which was not certified by peer review) is the author/funder, who has granted bioRxiv a license to display the preprint in perpetuity. It is made available under a CC-BY 4.0 International license.

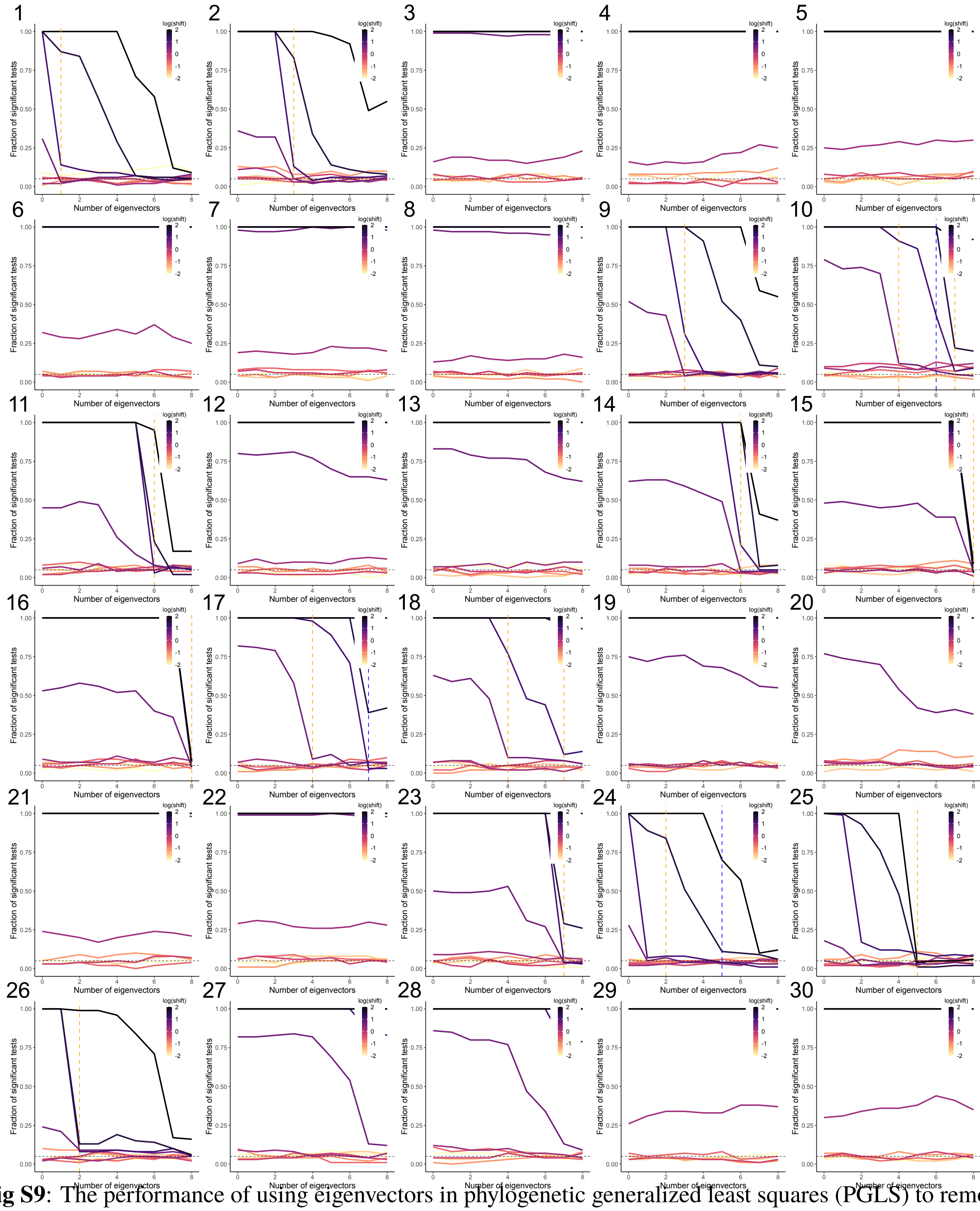

Fig S9: The performance of using eigenvectors in phylogenetic generalized least squares (PGLS) to remove phylogenetic confounding in a model with 16 tips related by a Yule tree with non-Brownian shifts in both predictor and outcome variables. The shift is simulated by adding a constant with varying size to the species that are descendants of the branch. The label of each panel corresponds to the label of the branch where the shift occurs on the tree in Fig. S7. The horizontal axis shows the number of eigenvectors included as covariates, and the vertical axis shows the fraction of tests that would be significant at the 0.05 level. The vertical orange dashed lines show the dimensions to which the branch concerned makes large positive contributions, and the vertical blue dashed lines show the dimensions to which the branch concerned makes large negative contributions.

bioRxiv preprint doi: https://doi.org/10.64898/2025.12.04.692436; this version posted December 5, 2025. The copyright holder for this preprint (which was not certified by peer review) is the author/funder, who has granted bioRxiv a license to display the preprint in perpetuity. It is made available under a CC-BY 4.0 International license.

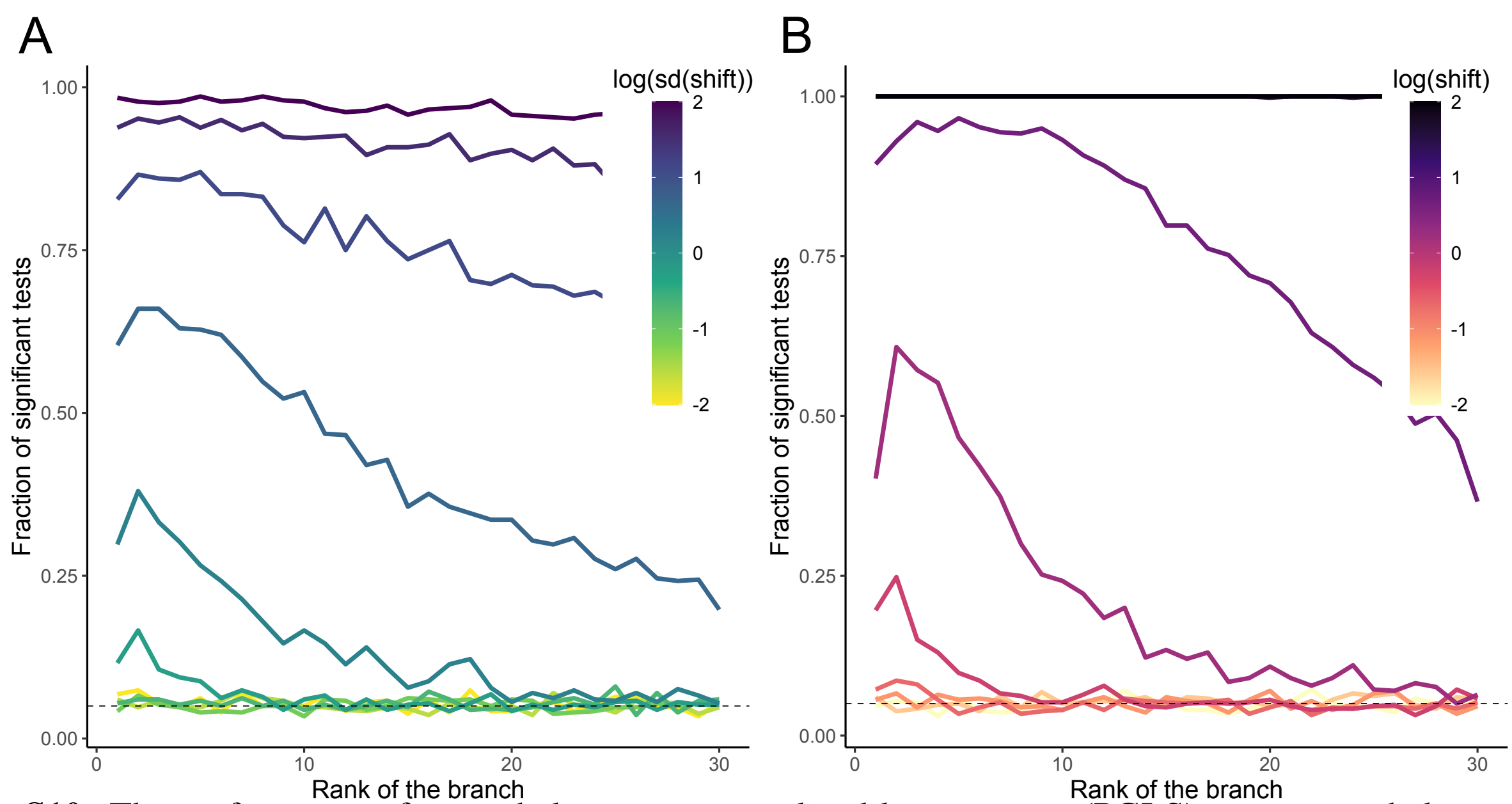

Fig S10: The performance of pure phylogenetic generalized least squares (PGLS) to remove phylogenetic confounding in models with 16 tips related by a Yule tree with a non-Brownian shift in both predictor and outcome variables. (A) The shift is simulated by adding a normal random variable with varying standard deviation to the species that are descendants of the branch. The horizontal axis shows the rank of the branches by overall contributions in decreasing order, and the vertical axis shows the fraction of tests that would be significant at the 0.05 level. (B) The shift is simulated by adding a constant with varying size to the species that are descendants of the branch. The horizontal axis shows the rank of the branches by overall contributions in decreasing order, and the vertical axis shows the fraction of tests that would be significant at the 0.05 level.

bioRxiv preprint doi: https://doi.org/10.64898/2025.12.04.692436; this version posted December 5, 2025. The copyright holder for this preprint (which was not certified by peer review) is the author/funder, who has granted bioRxiv a license to display the preprint in perpetuity. It is made available under a CC-BY 4.0 International license.

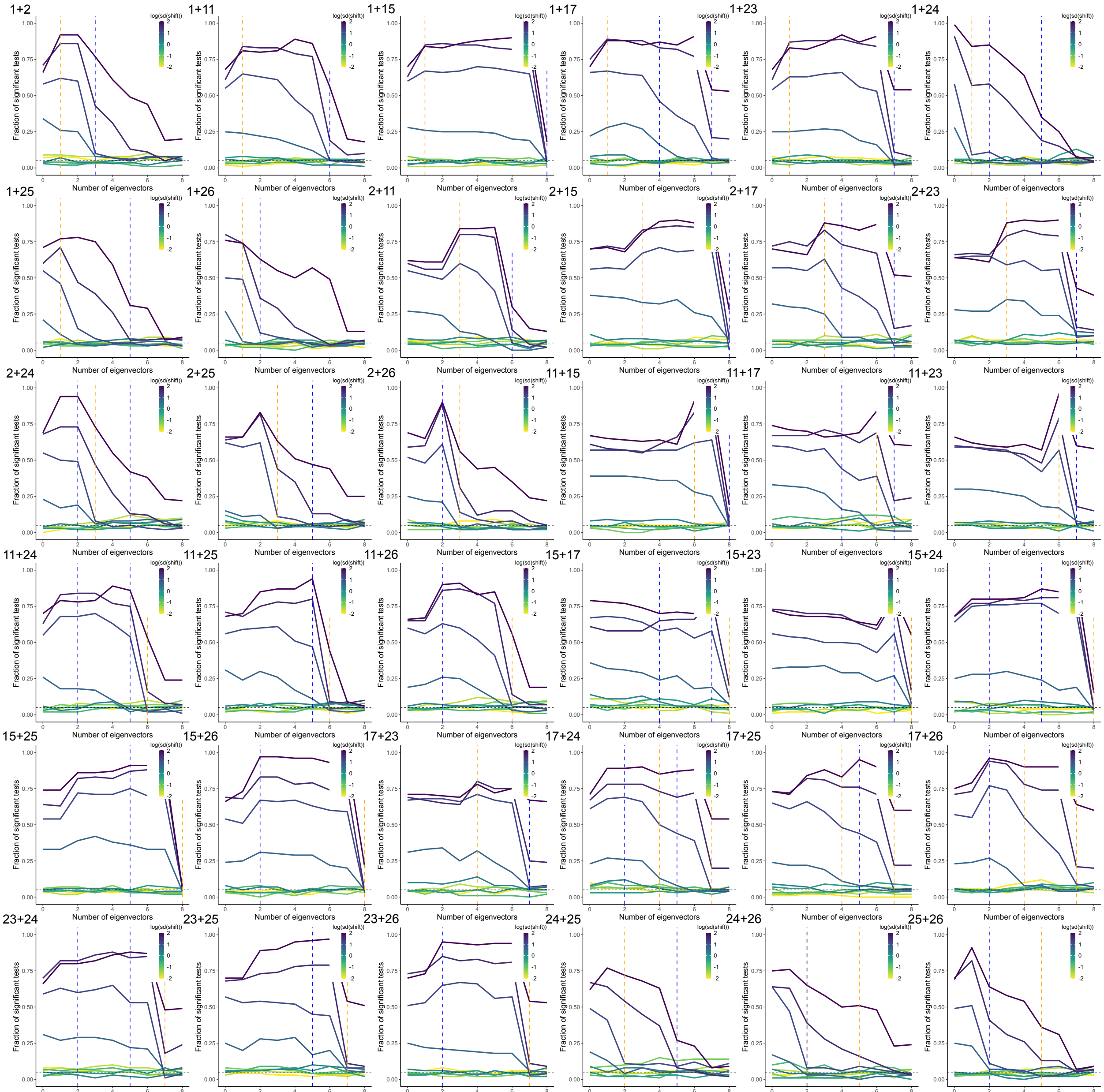

Fig S11: The performance of using eigenvectors in phylogenetic generalized least squares (PGLS) to remove phylogenetic confounding in a model with 16 tips related by a Yule tree with two non-Brownian shifts in both predictor and outcome variables. The shifts are simulated by adding a random normal variable with varying standard deviation to the species that are descendants of the branch. The label of each panel corresponds to the labels of the two branches where the shifts occur on the tree in Fig. S7. The horizontal axis shows the number of eigenvectors included as covariates, and the vertical axis shows the fraction of tests that would be significant at the 0.05 level. The vertical orange dashed lines show the dimensions to which the first branch concerned makes large contributions, and the vertical blue dashed lines show the dimensions to which the second branch concerned makes large contributions.

bioRxiv preprint doi: https://doi.org/10.64898/2025.12.04.692436; this version posted December 5, 2025. The copyright holder for this preprint (which was not certified by peer review) is the author/funder, who has granted bioRxiv a license to display the preprint in perpetuity. It is made available under a CC-BY 4.0 International license.

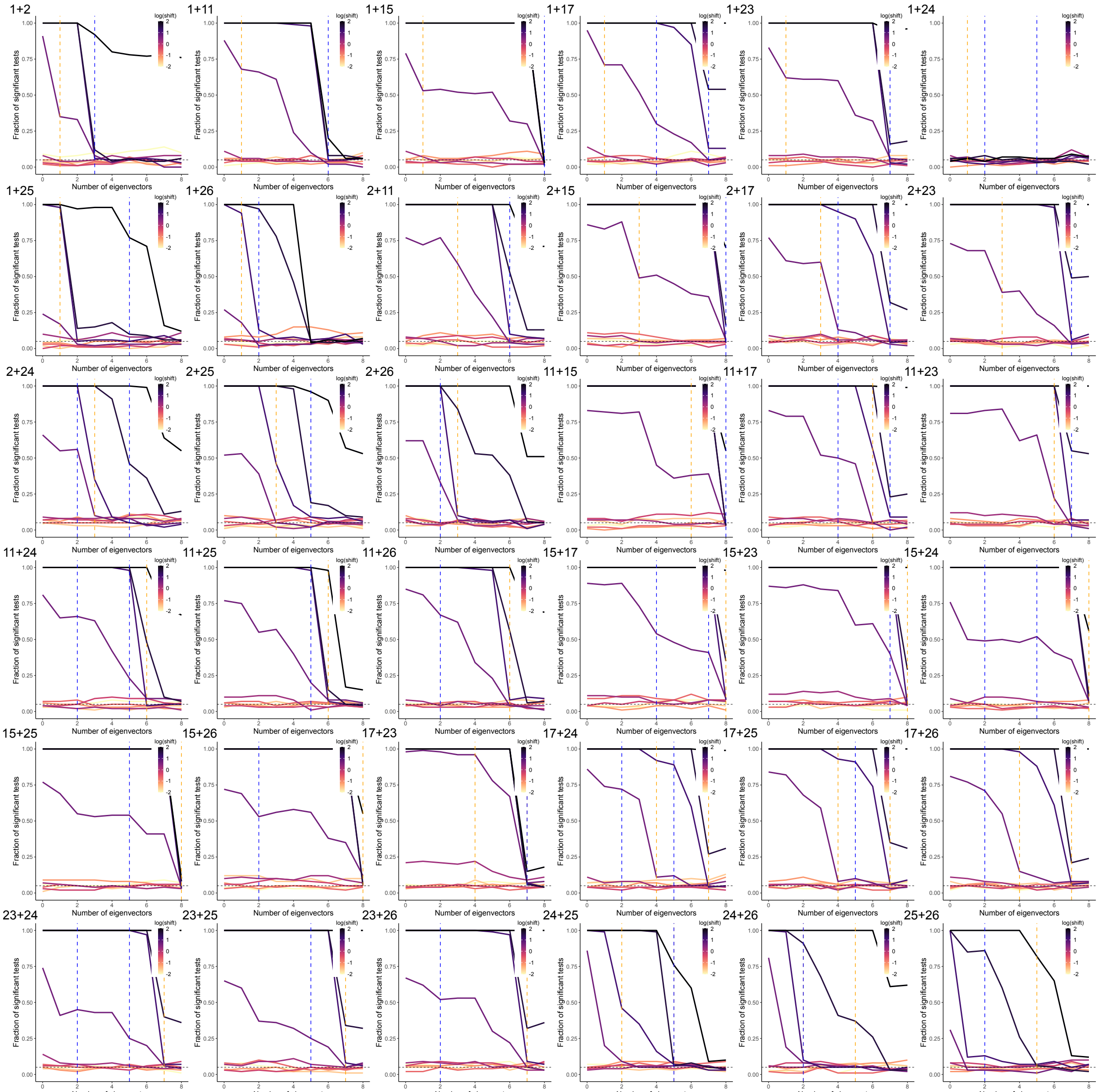

Fig S12: The performance of using eigenvectors in phylogenetic generalized least squares (PGLS) to remove phylogenetic confounding in a model with 16 tips related by a Yule tree with two non-Brownian shifts in both predictor and outcome variables. The shifts are simulated by adding a constant with varying size to the species that are descendants of the branch. The label of each panel corresponds to the labels of the two branches where the shifts occur on the tree in Fig. S7. The horizontal axis shows the number of eigenvectors included as covariates, and the vertical axis shows the fraction of tests that would be significant at the 0.05 level. The vertical orange dashed lines show the dimensions to which the first branch concerned makes large contributions, and the vertical blue dashed lines show the dimensions to which the second branch concerned makes large contributions.

bioRxiv preprint doi: https://doi.org/10.64898/2025.12.04.692436; this version posted December 5, 2025. The copyright holder for this preprint (which was not certified by peer review) is the author/funder, who has granted bioRxiv a license to display the preprint in perpetuity. It is made available under a CC-BY 4.0 International license.

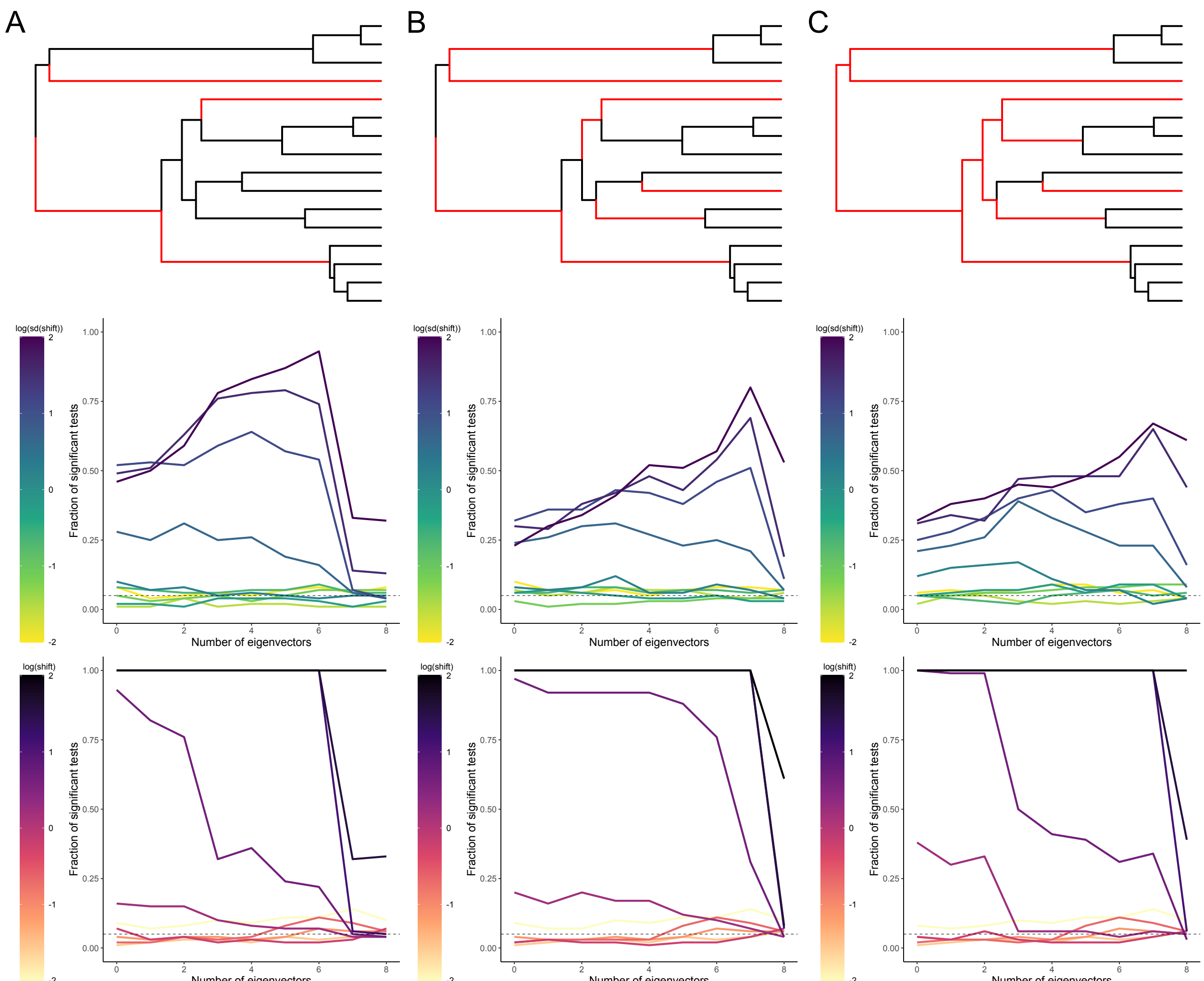

Fig S13: The performance of using eigenvectors in phylogenetic generalized least squares (PGLS) to remove phylogenetic confounding in a model with 16 tips related by a Yule tree with multiple non-Brownian shifts in both predictor and outcome variables. The top panels show the branches where the shifts occur in red. The middle panels show the performance of using eigenvectors in PGLS to remove phylogenetic confounding in the model, where the shifts are simulated by adding a normal random variable with varying standard deviation to the species that are descendants of the branch. The horizontal axis shows the number of eigenvectors included as covariates, and the vertical axis shows the fraction of tests that would be significant at the 0.05 level. The bottom panels show the performance of using eigenvectors in PGLS to remove phylogenetic confounding in the model, where the shifts are simulated by adding a constant of varying size to the species that are descendants of the branch. The horizontal axis shows the number of eigenvectors included as covariates, and the vertical axis shows the fraction of tests that would be significant at the 0.05 level. (A) There are 4 shifts on the tree. (B) There are 8 shifts on the tree. (C) There are 12 shifts on the tree.

bioRxiv preprint doi: https://doi.org/10.64898/2025.12.04.692436; this version posted December 5, 2025. The copyright holder for this preprint (which was not certified by peer review) is the author/funder, who has granted bioRxiv a license to display the preprint in perpetuity. It is made available under a CC-BY 4.0 International license.

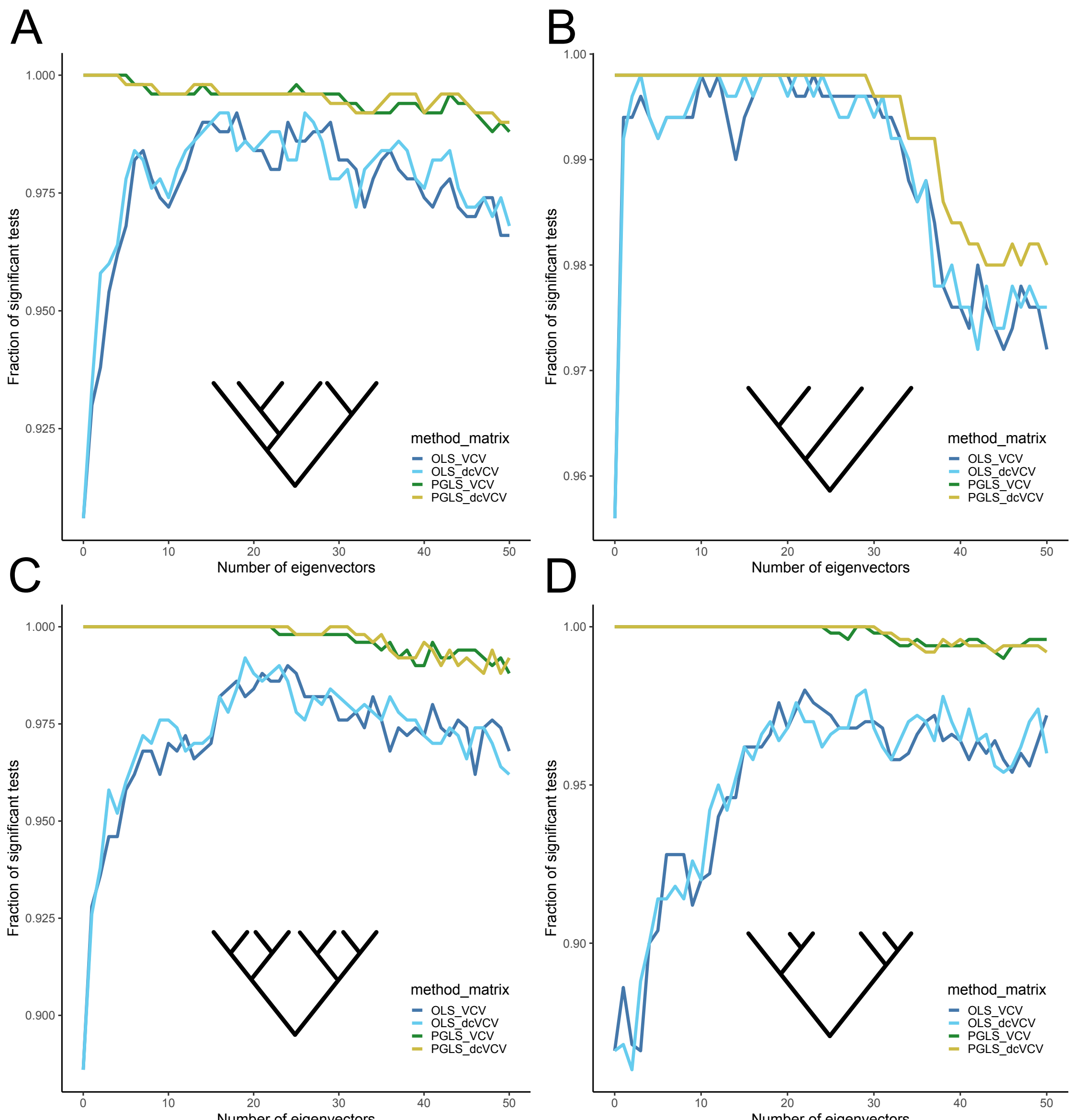

Fig S14: The performance of using eigenvectors of non-centered and double-centered phylogenetic variance-covariance matrices (C and $\breve{C}$) in ordinary least squares (OLS) and phylogenetic generalized least squares (PGLS) for different types of ultrametric trees when the true regression coefficient for x, β, is 0.5. (A) Performance of OLS and PGLS with eigenvectors of C and $\breve{C}$ in models related by a Yule tree of 128 species. For simplicity, a Yule tree with only 6 tips is shown. The horizontal axis shows the number of eigenvectors included as covariates, and the vertical axis shows the fraction of tests that would be significant at the 0.05 level. (B) Performance of OLS and PGLS with eigenvectors of C and $\breve{C}$ in models related by a caterpillar tree of 128 species. For simplicity, a caterpillar tree with only 4 tips is shown. The horizontal axis shows the number of eigenvectors included as covariates, and the vertical axis shows the fraction of tests that would be significant at the 0.05 level. The lines for PGLS_VCV and PGLS_dcVCV are overlapping. (C) Performance of OLS and PGLS with eigenvectors of C and $\breve{C}$ in models related by a fully balanced tree of 128 species. For simplicity, a fully balanced tree with only 8 tips is shown. The horizontal axis shows the number of eigenvectors included as covariates, and the vertical axis shows the fraction of tests that would be significant at the 0.05 level. (D) Performance of OLS and PGLS with eigenvectors of C and $\breve{C}$ in models related by a coalescent tree of 128 species. For simplicity, a coalescent tree with only 6 tips is shown. The horizontal axis shows the number of eigenvectors included as covariates, and the vertical axis shows the fraction of tests that would be significant at the 0.05 level.

bioRxiv preprint doi: https://doi.org/10.64898/2025.12.04.692436; this version posted December 5, 2025. The copyright holder for this preprint (which was not certified by peer review) is the author/funder, who has granted bioRxiv a license to display the preprint in perpetuity. It is made available under a CC-BY 4.0 International license.

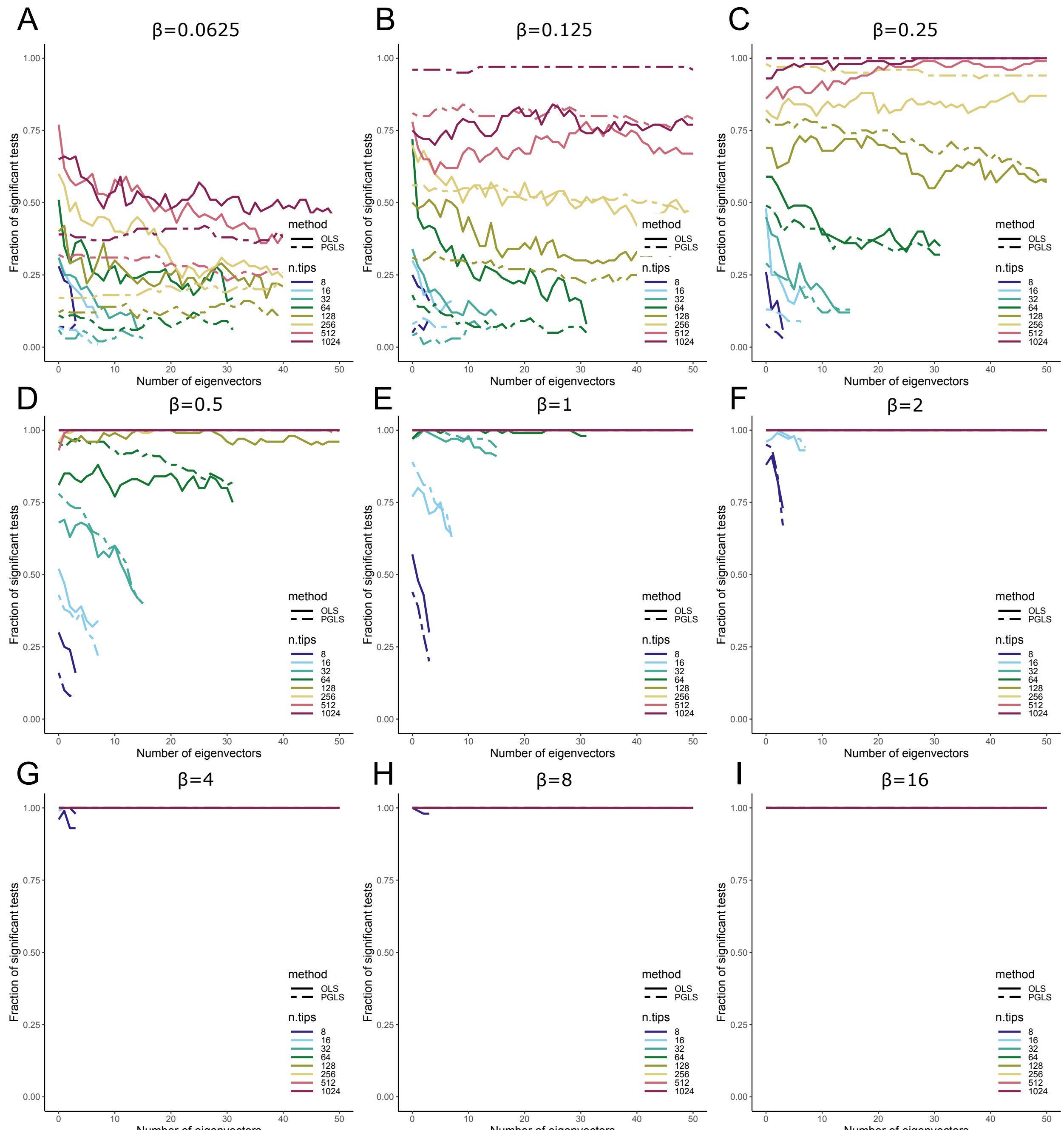

Fig S15: The recall after adding eigenvectors in ordinary least squares (OLS) and phylogenetic generalized least squares (PGLS) for Yule trees of varying numbers of species and different non-zero values of the true regression coefficient for x, β. The horizontal axes show the number of eigenvectors included as covariates, and the vertical axes show the fraction of tests that would be significant at the 0.05 level. (A) $\beta = 0.0625$. (B) $\beta = 0.125$. (C) $\beta = 0.25$. (D) $\beta = 0.5$. (E) $\beta = 1$. (F) $\beta = 2$. (G) $\beta = 4$. (H) $\beta = 8$. (I) $\beta = 16$.

bioRxiv preprint doi: https://doi.org/10.64898/2025.12.04.692436; this version posted December 5, 2025. The copyright holder for this preprint (which was not certified by peer review) is the author/funder, who has granted bioRxiv a license to display the preprint in perpetuity. It is made available under a CC-BY 4.0 International license.

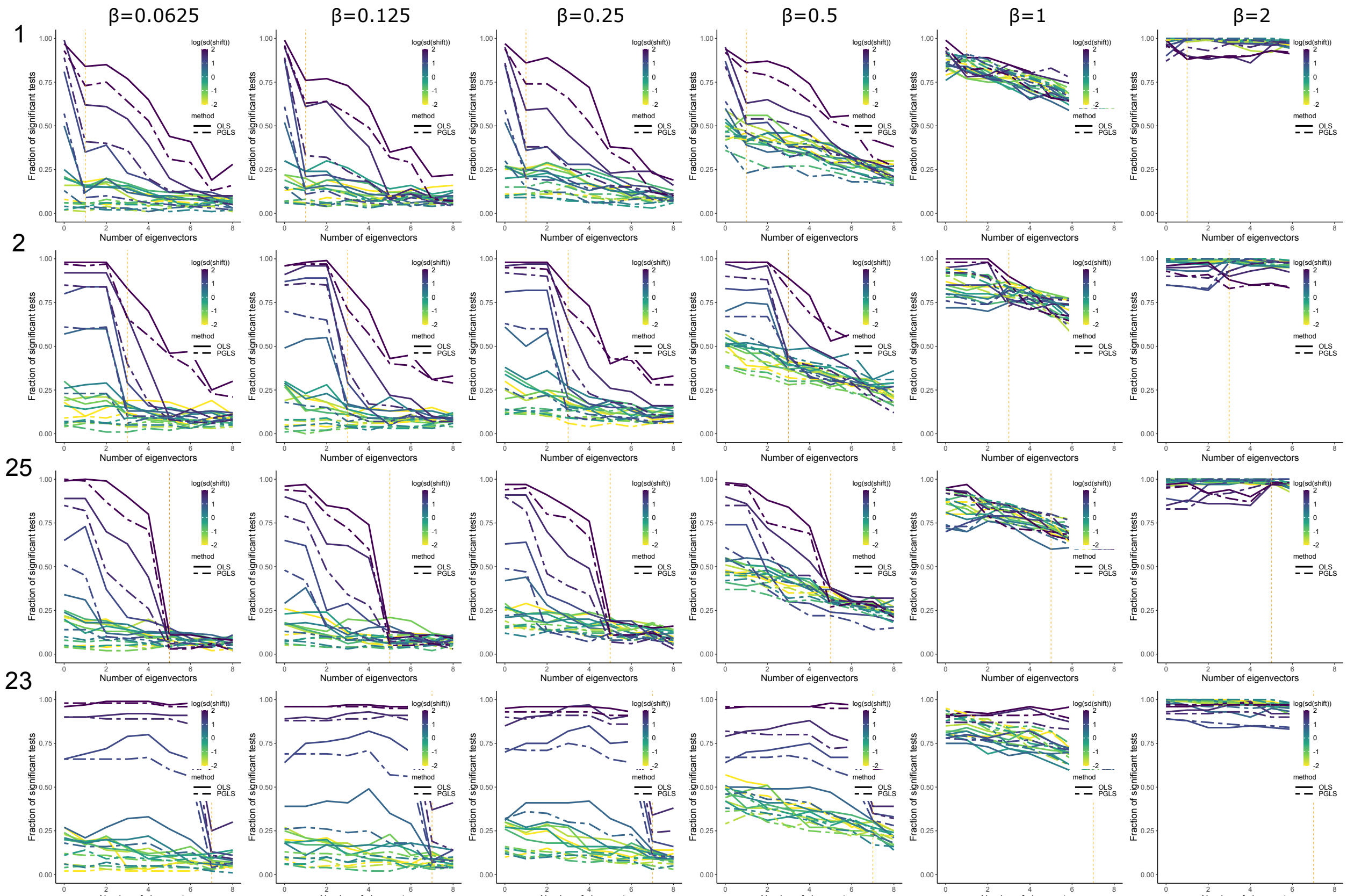

Fig S16: The recall after adding eigenvectors in ordinary least squares (OLS) and phylogenetic generalized least squares (PGLS) in a model with 16 tips related by a Yule tree with a non-Brownian shift in both predictor and outcome variables for different non-zero values of the true regression coefficient for x, β. The shift is simulated by adding a normal random variable with varying standard deviation to the species that are descendants of the branch. The horizontal axes show the number of eigenvectors included as covariates, and the vertical axes show the fraction of tests that would be significant at the 0.05 level. The numbers in front of each row indicate the labels of the branches where the shifts occur on the tree in Fig. S7, and each column corresponds to a different β value in 0.0625, 0.125, 0.25, 0.5, 1, and 2. The vertical orange dashed lines show the dimensions to which the branch concerned makes large contributions.

bioRxiv preprint doi: https://doi.org/10.64898/2025.12.04.692436; this version posted December 5, 2025. The copyright holder for this preprint (which was not certified by peer review) is the author/funder, who has granted bioRxiv a license to display the preprint in perpetuity. It is made available under a CC-BY 4.0 International license.

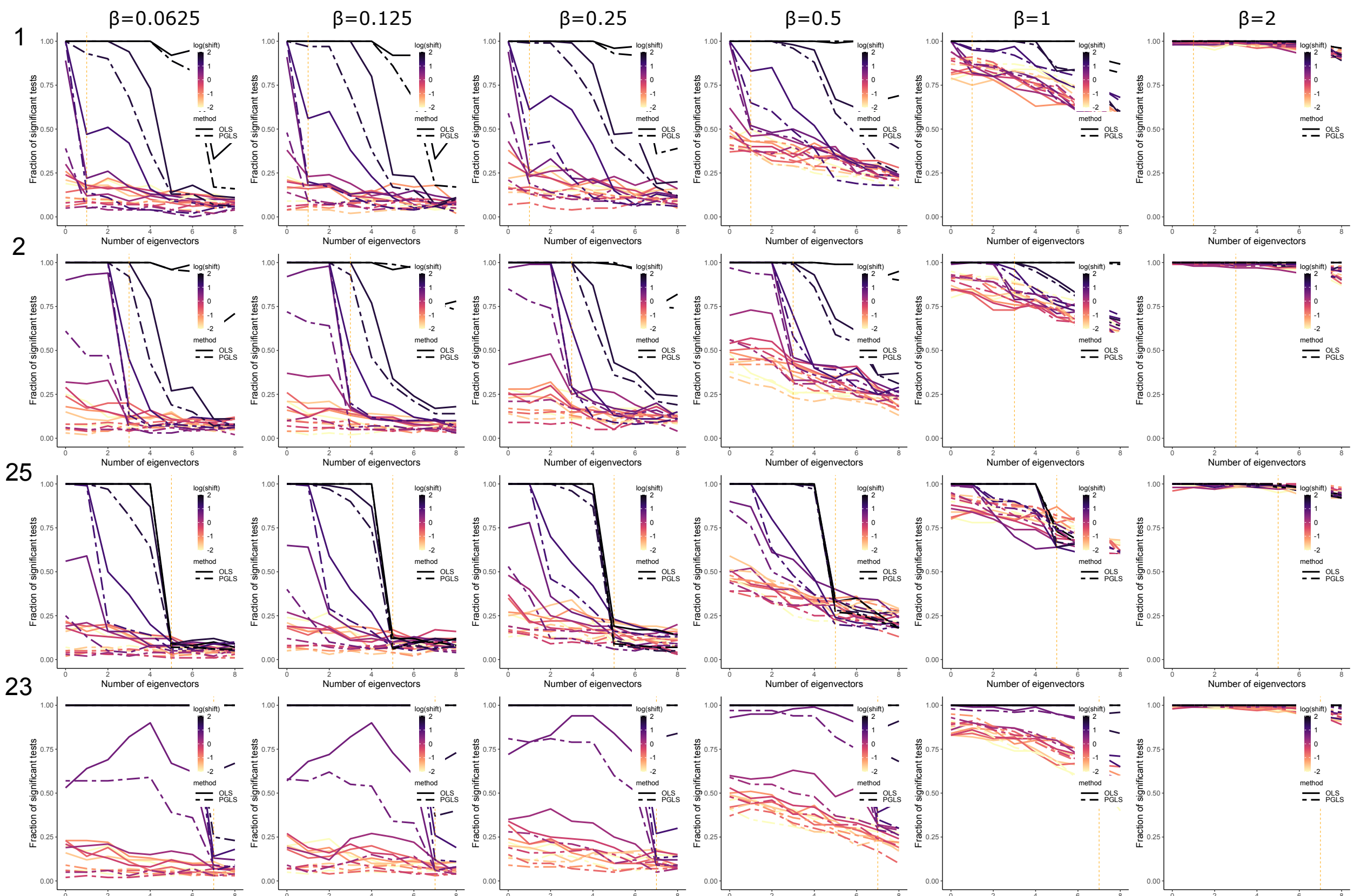

Fig S17: The recall after adding eigenvectors in ordinary least squares (OLS) and phylogenetic generalized least squares (PGLS) in a model with 16 tips related by a Yule tree with a non-Brownian shift in both predictor and outcome variables for different non-zero values of the true regression coefficient for x, β. The shift is simulated by adding a constant with varying size to the species that are descendants of the branch. The horizontal axes show the number of eigenvectors included as covariates, and the vertical axes show the fraction of tests that would be significant at the 0.05 level. The numbers in front of each row indicate the labels of the branches where the shifts occur on the tree in Fig. S7, and each column corresponds to a different β value in 0.0625, 0.125, 0.25, 0.5, 1, and 2. The vertical orange dashed lines show the dimensions to which the branch concerned makes large contributions.

bioRxiv preprint doi: https://doi.org/10.64898/2025.12.04.692436; this version posted December 5, 2025. The copyright holder for this preprint (which was not certified by peer review) is the author/funder, who has granted bioRxiv a license to display the preprint in perpetuity. It is made available under a CC-BY 4.0 International license.

References

[1] de Vienne, D. M., G. Aguileta, and S. Ollier. 2011. Euclidean nature of phylogenetic distance matrices. Systematic biology 60:826–832.

[2] Diniz-Filho, J. A. F., C. E. R. d. Sant'Ana, and L. M. Bini. 1998. An eigenvector method for estimating phylogenetic inertia. Evolution 52:1247–1262.

[3] Fan, C., N. Mancuso, and C. W. Chiang. 2022. A genealogical estimate of genetic relationships. The American Journal of Human Genetics 109:812–824.

[4] Ho, L. S. T. and C. Ané. 2014. A linear-time algorithm for gaussian and non-gaussian trait evolution models. Systematic biology 63:397–408.

[5] Ho, L. S. T., C. Ane, R. Lachlan, K. Tarpinian, R. Feldman, Q. Yu, W. van der Bijl, J. Maspons, R. Vos, and M. Ho. 2016. Package 'phylolm'. URL: https://CRAN.R-project.org/package=phylolm.

[6] McVean, G. 2009. A genealogical interpretation of principal components analysis. PLoS genetics 5:e1000686.

[7] Schraiber, J. G., M. D. Edge, and M. Pennell. 2024. Unifying approaches from statistical genetics and phylogenetics for mapping phenotypes in structured populations. PLoS biology 22:e3002847.

[8] Weyl, H. 1912. Das asymptotische verteilungsgesetz der eigenwerte linearer partieller differentialgleichungen (mit einer anwendung auf die theorie der hohlraumstrahlung). Mathematische Annalen 71:441–479.

[9] Yang, J., B. Benyamin, B. P. McEvoy, S. Gordon, A. K. Henders, D. R. Nyholt, P. A. Madden, A. C. Heath, N. G. Martin, G. W. Montgomery, et al. 2010. Common snps explain a large proportion of the heritability for human height. Nature genetics 42:565–569.
